# Supplementary material for: A systematic machine learning and data type comparison yields metagenomic predictors of infant age, sex, breastfeeding, antibiotic usage, country of origin, and delivery type
Source: PLoS Comput Biol. 2020 May 11;16(5):e1007895. doi: 10.1371/journal.pcbi.1007895 (PMC7241849; doi:10.1371/journal.pcbi.1007895)
Supplement: S1 Text. An overview of our machine learning toolset and additional technical notes on outliers in our results — (PDF) [file pcbi.1007895.s001.pdf]

1. A brief overview of our selected machine learning tools

We employ a range of modeling strategies in our manuscript. Here, we describe the broad characteristics, similarities, and differences between them.

The simplest method we use is an Elastic Net (EN). An EN is a combination of Ridge and Lasso regression -- it is a linear model designed to aid in variable selection, where highly-outcome-associated features (e.g. species, pathways, or genes) are identified among a large set. Given their nature, ENs are only capable of capturing linear-relationships between dependent and independent variables, meaning that while their results are at times easier to interpret biologically and statistically than other “black-box” methods, they can potentially overlook valid non-linear structures within the data.

We additionally employed Random Forests (RFs), ensemble learning methods that are popular in microbiome analyses. Each random forest is a combination of decision trees fit to the data, where each the nodes of each tree represent classes or outcomes, and the branch points represent features in the data that distinguish different classes or outcomes. Individual decision trees are fit on a subsample of the data -- the “Forest” results from the combination (weighted averaging) of these individual trees. RFs are able to capture non-linear effects, it is difficult to carry out statistical inference on their output due to their “black box” nature (e.g., information on how increasing a certain variable modulates the dependent variable is not as easily attainable as for Elastic Nets).

Gradient-Boosted-Machines (GBMs) are another ensemble learning approach that we use. GBMs leverage “gradient boosting” -- often applied to decision trees -- where “weak-learners” (poorly performing trees) are optimized for increased performance by, for a given tree, increasing the weights on difficult-to-classify/predict features in the training set and lowering the weights on the easy-to-classify outcomes. The next tree is built with these weights, therefore resulting in a model sensitive to variation across outcomes. Like an RF, GBMs too are often treated as black boxes, so despite being able to capture non-linear effects, they can also be difficult to interpret (e.g., information on how increasing a certain variable modulates the dependent variable is not as easily attainable as for Elastic Nets).

We additionally used 3 different Support Vector Machines (SVMs), with a linear, polynomial, and radial kernel. SVMs attempt to project data into an N-dimensional space where it can be spatially separated into groups. The kernel in question corresponds to how the data are projected into this new coordinate plane; depending on the kernel, an SVM can capture non-linear effects. Again, inference on the individual variables is not easily attainable.
