# Supplementary material for: A systematic machine learning and data type comparison yields metagenomic predictors of infant age, sex, breastfeeding, antibiotic usage, country of origin, and delivery type
Source: PLoS Comput Biol. 2020 May 11;16(5):e1007895. doi: 10.1371/journal.pcbi.1007895 (PMC7241849; doi:10.1371/journal.pcbi.1007895)
Supplement: S2 Text — (PDF) [file pcbi.1007895.s002.pdf]

2. On the negative prediction performances for some of our algorithms

Some algorithms had negative prediction performances (e.g. AUC) for a range of phenotypes. For example, there is indeed a negative performance for R2_test for ElasticNet, and what is interesting to see is that this negative score is driven by a single fold (fold 3) that performed extremely poorly.

It is also interesting to note that this anomaly is found on the same fold for ElasticNet 2, SVM Linear and SVM Polynomial of degree 2, but did not prevent the tree based models (random forest, gradient boosted machine), the KNN and the SVM Radial from performing well. (The SVM Radial performed poorly on the first fold, but not the third one).

This negative performance is only found on the testing set, not on the training set, so the model learned correctly but generalized poorly. What is also interesting to observe is that by looking at the training results and the hyperparameters that were selected, nothing initially suggested that the model trained on this fold would generalize worse than the others: both the training performance and the hyperparameters are similar.

We think there are two main reasons why this model generalized poorly:

1-It is possible that an outlier in the testing set was predicted with a huge error, which is driving this hugely negative R-Squared score. One way to test it would be to leave out different subsets of the testing set when computing the R-Squared until this negative score disappears to isolate the samples responsible for it. We could follow up with an analysis as to what makes these samples so unpredictable by the linear regression.

2-The problem is not on the testing set but on the training set. This seems unlikely since this training set shares 89% of its data with the other training sets, but it is possible that this specific combination lead to a poor learning of the regression weights and strong overfitting. One way to test for that would be to build a correlation matrix between the vector of the weights for each of the 10 models (one for each fold) and to see if the weights learned on the 3rd model differ significantly from the others.

That being said, figuring out why exactly this specific model performed poorly is beyond the scope of this paper. We were only interested in figuring out which model would yield the most robust prediction and we think that the global negative performance of the ElasticNet model sends the right message: it is not a suitable choice for this specific prediction task.

The NAs performance values for SVM solely come from the predictions using genes. We extracted the genes from the 10 most significant CAGs. For ElasticNet, we took the best 10 CAGs selected by the elastic net. For the GBM, we took the best 10 CAGs selected by the gradient boosted machine. However for the SVM, there is to our knowledge no way to tell which CAGs were the most significant. We could therefore not extract genes, and this is the reason why we used NAs as placeholders for SVMs. This is also true for the Naive Bayes algorithm. Only the elastic nets, the random forests and the gradient boosted machines allowed us to select significant cags and to extract genes from them.

An alternative would have been to use the genes selected by a different algorithm (e.g GBM) to

predict the target phenotypes using SVMs and Naive Bayes. Out of consistency we did not do it. Considering the general performance of SVMs and Naive Bayes across the different predictors and targets, we think it is unlikely that they would have performed significantly better than other algorithms using genes as predictors.
