# Supplementary figures and images for: A systematic machine learning and data type comparison yields metagenomic predictors of infant age, sex, breastfeeding, antibiotic usage, country of origin, and delivery type
Source: PLoS Comput Biol. 2020 May 11;16(5):e1007895. doi: 10.1371/journal.pcbi.1007895 (PMC7241849; doi:10.1371/journal.pcbi.1007895)

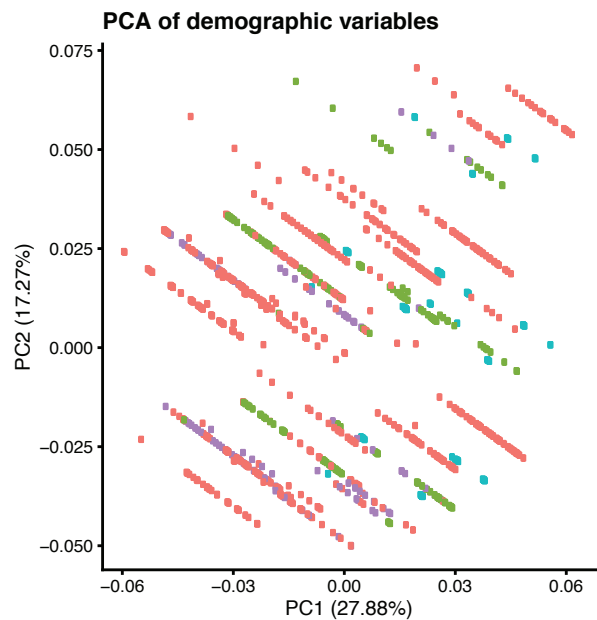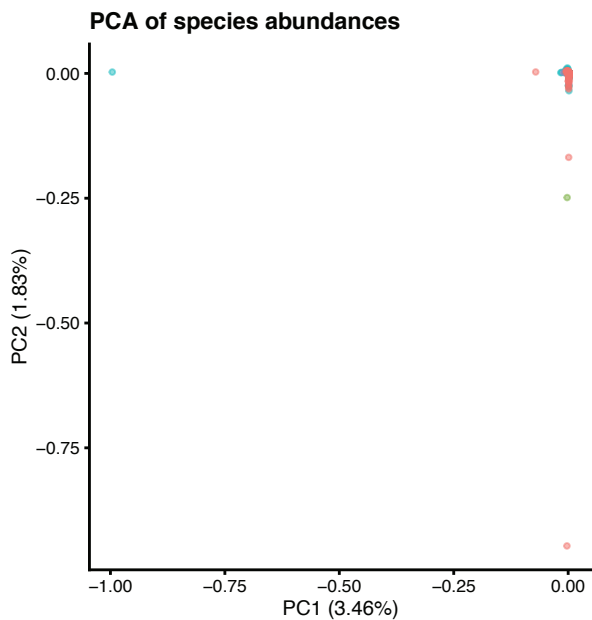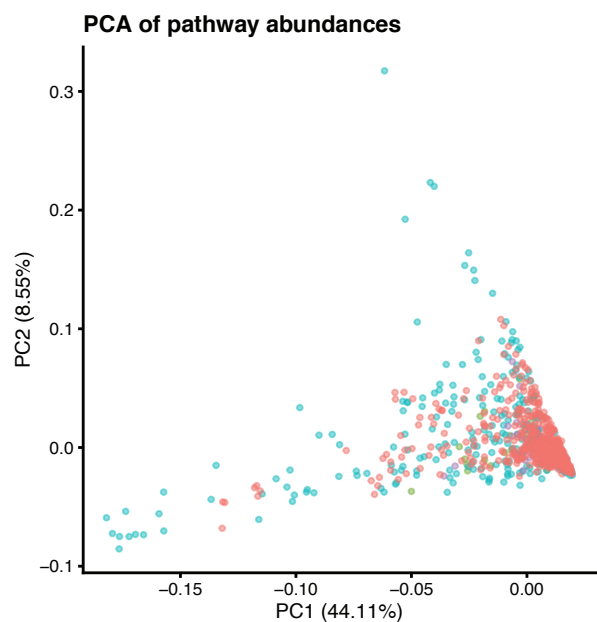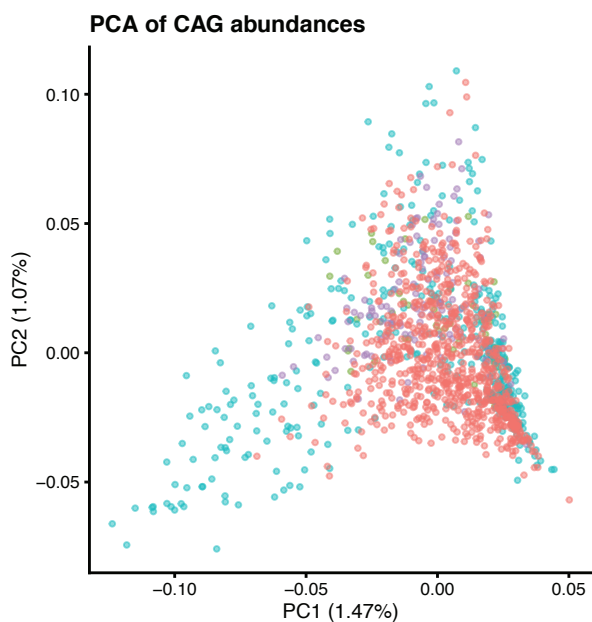

- Vatanen\_2016
- Yassour\_2016
- Backhed\_2015
- Kostic\_2015

Supplement: S1 Fig — (PDF) [file pcbi.1007895.s011.pdf]

Number of genes in each CAG

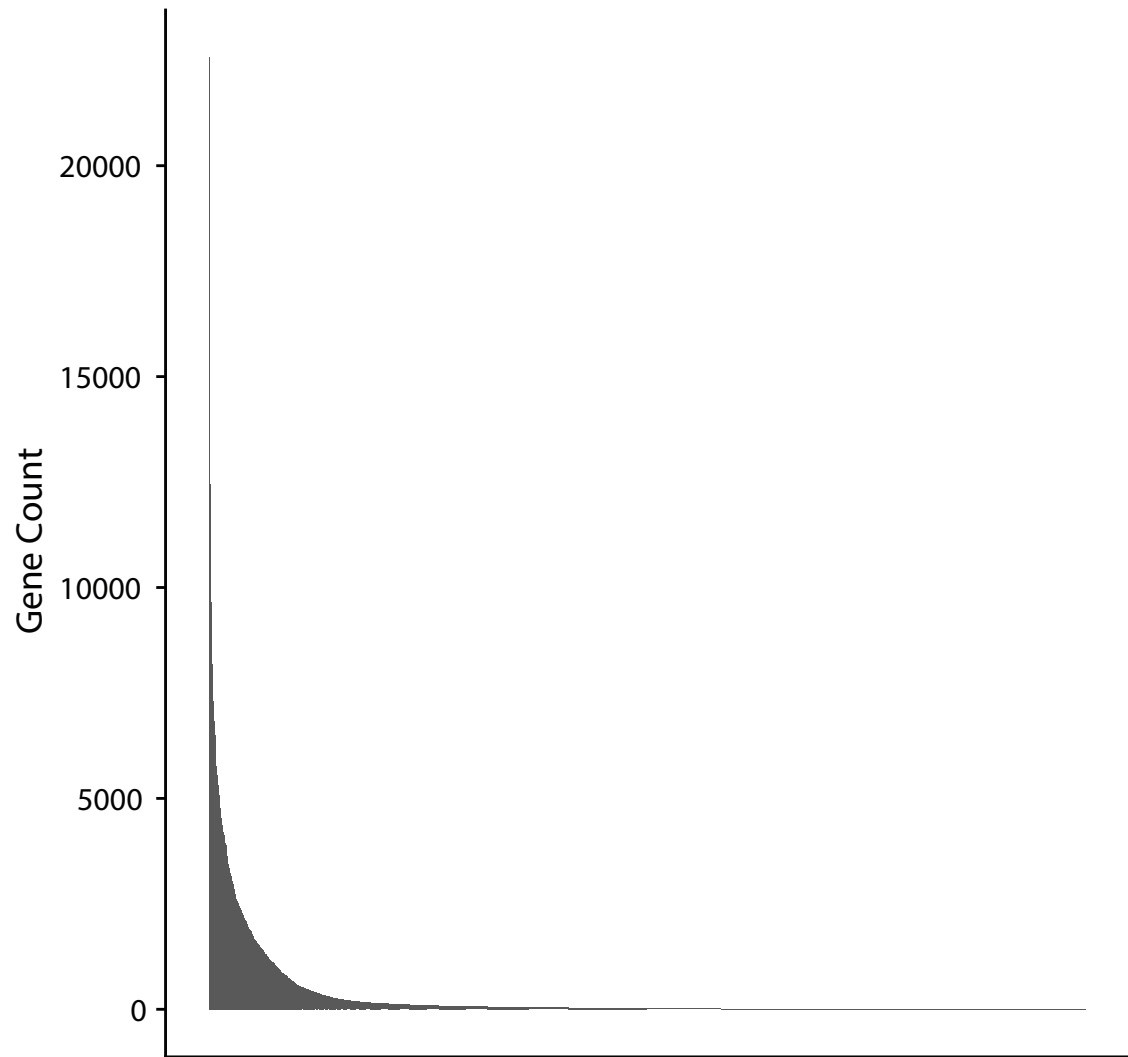

Quantiles

|    |     |     |     |       |
|----|-----|-----|-----|-------|
| 0% | 25% | 50% | 75% | 100%  |
| 2  | 5   | 23  | 93  | 22563 |

Supplement: S2 Fig — (PDF) [file pcbi.1007895.s012.pdf]

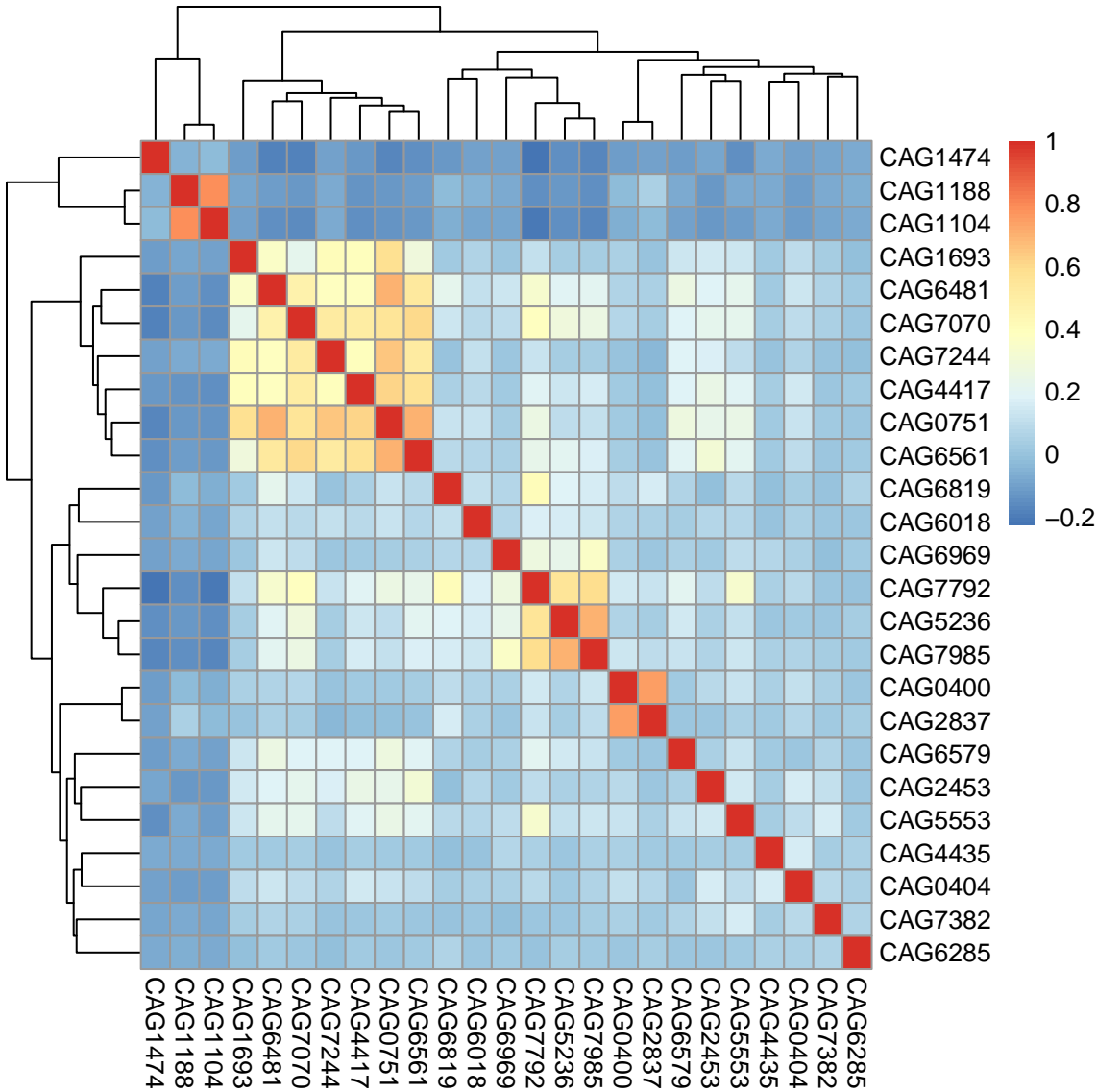

Supplement: S4 Fig — (PDF) [file pcbi.1007895.s014.pdf]

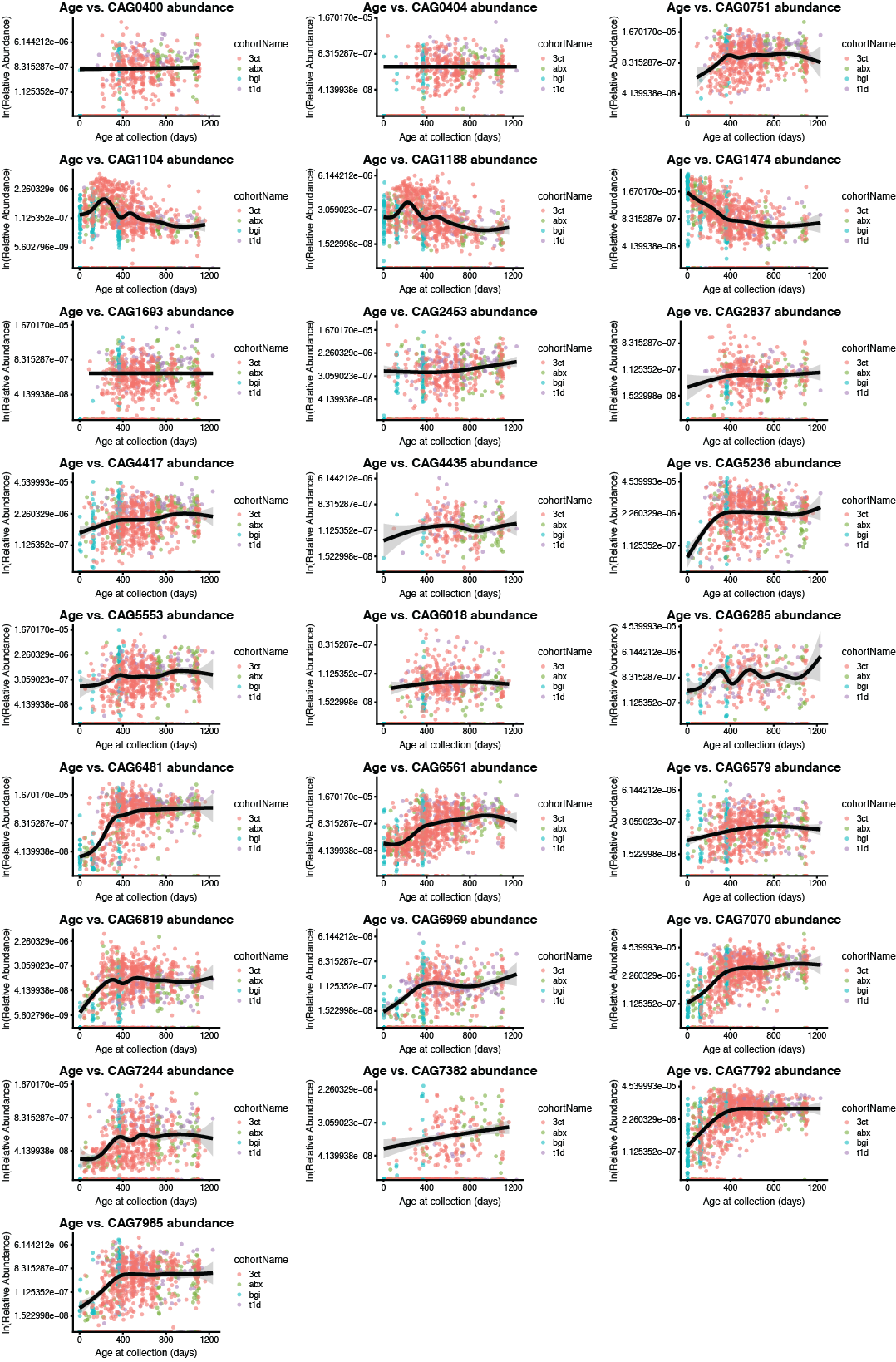

Supplement: S6 Fig — (PNG) [file pcbi.1007895.s016.png]

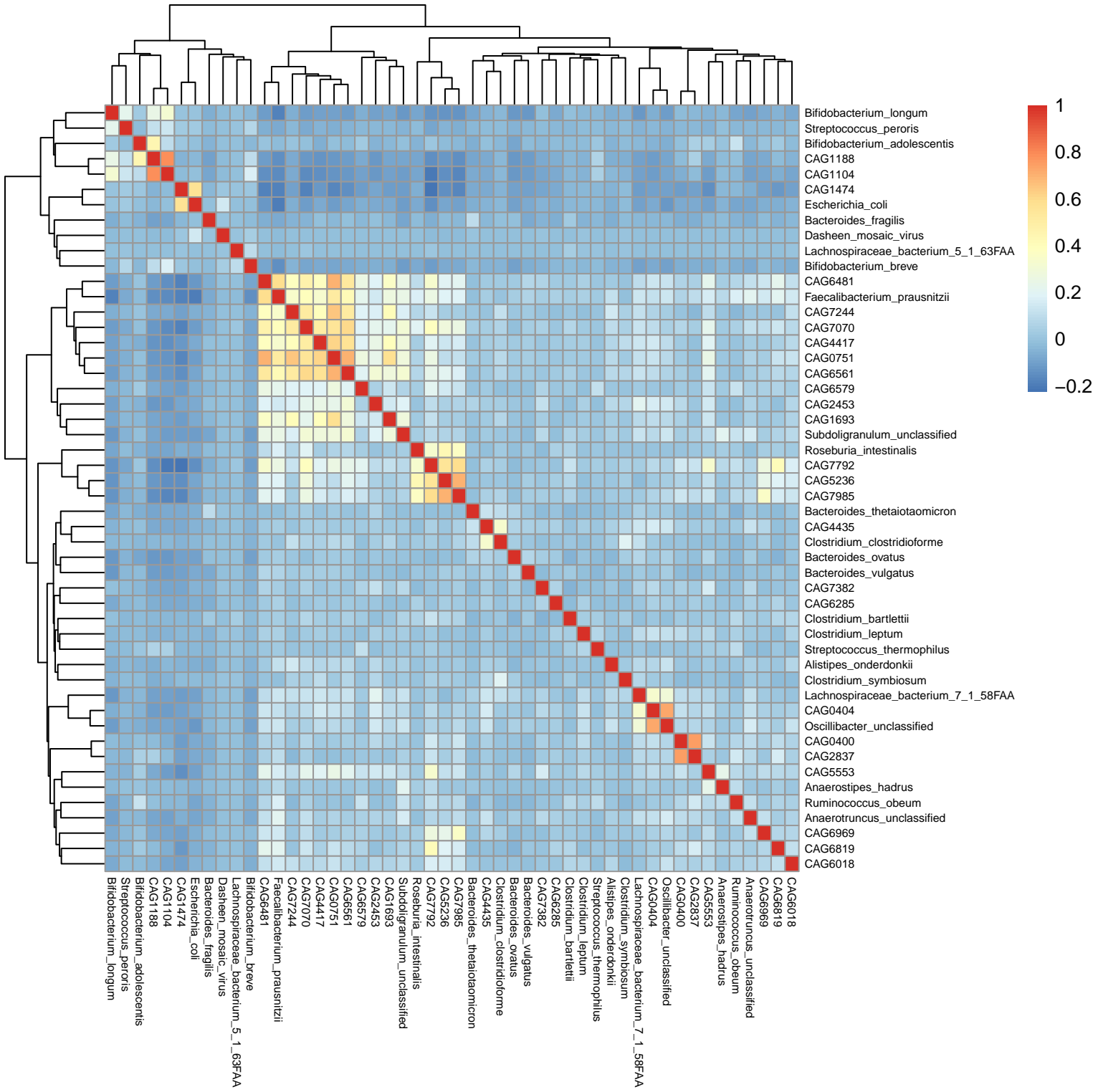

Supplement: S8 Fig — (PDF) [file pcbi.1007895.s018.pdf]
