# Supplementary material for: A systematic machine learning and data type comparison yields metagenomic predictors of infant age, sex, breastfeeding, antibiotic usage, country of origin, and delivery type
Source: PLoS Comput Biol. 2020 May 11;16(5):e1007895. doi: 10.1371/journal.pcbi.1007895 (PMC7241849; doi:10.1371/journal.pcbi.1007895)
Supplement: S3 Fig — (PDF) [file pcbi.1007895.s013.pdf]

Nested CV: Outer CV=10 folds, Inner CV=5 folds

N=100	Outer CV folds 1											
N=10	Train		N=90	Inner CV folds 1	N=90	Inner CV folds 2	N=90	Inner CV folds 3	N=90	Inner CV folds 4	N=90	Inner CV folds 5
N=10	Train		N=18	Train_train	N=18	Train_train	N=18	Train_train	N=18	Train_train	N=18	Train_validation
N=10	Train		N=18	Train_train	N=18	Train_train	N=18	Train_train	N=18	Train_validation	N=18	Train_train
N=10	Train	zoom on train =>	N=18	Train_train +	N=18	Train_train +	N=18	Train_valida +	N=18	Train_train +	N=18	Train_train
N=10	Train		N=18	Train_train	N=18	Train_validation	N=18	Train_train	N=18	Train_train	N=18	Train_train
N=10	Train		N=18	Train_validation	N=18	Train_train	N=18	Train_train	N=18	Train_train	N=18	Train_train
N=10	Train											
N=10	Test											

N=100	Outer CV folds 2											
N=10	Train		N=90	Inner CV folds 1	N=90	Inner CV folds 2	N=90	Inner CV folds 3	N=90	Inner CV folds 4	N=90	Inner CV folds 5
N=10	Train		N=18	Train_train	N=18	Train_train	N=18	Train_train	N=18	Train_train	N=18	Train_validation
N=10	Train		N=18	Train_train	N=18	Train_train	N=18	Train_train	N=18	Train_validation	N=18	Train_train
N=10	Train	zoom on train =>	N=18	Train_train +	N=18	Train_train +	N=18	Train_valida +	N=18	Train_train +	N=18	Train_train
N=10	Train		N=18	Train_train	N=18	Train_validation	N=18	Train_train	N=18	Train_train	N=18	Train_train
N=10	Train		N=18	Train_validation	N=18	Train_train	N=18	Train_train	N=18	Train_train	N=18	Train_train
N=10	Test											
N=10	Train											

.
.
.

N=100	Outer CV folds 10											
N=10	Test		N=90	Inner CV folds 1	N=90	Inner CV folds 2	N=90	Inner CV folds 3	N=90	Inner CV folds 4	N=90	Inner CV folds 5
N=10	Train		N=18	Train_train	N=18	Train_train	N=18	Train_train	N=18	Train_train	N=18	Train_validation
N=10	Train		N=18	Train_train	N=18	Train_train	N=18	Train_train	N=18	Train_validation	N=18	Train_train
N=10	Train	zoom on train =>	N=18	Train_train +	N=18	Train_train +	N=18	Train_valida +	N=18	Train_train +	N=18	Train_train
N=10	Train		N=18	Train_train	N=18	Train_validation	N=18	Train_train	N=18	Train_train	N=18	Train_train
N=10	Train		N=18	Train_validation	N=18	Train_train	N=18	Train_train	N=18	Train_train	N=18	Train_train
N=10	Train											
N=10	Train											

Merge the predictions on the 10 testing set folds to obtain the full testing set predictions, one for each sample (although not every prediction comes from the same model (parameter and hyperparameters).
10 different models are used to predict on the samples as testing sets. Then use the full 100 predictions to evaluate the testing performance of the model.

N=100	**Outer CV folds 0**											
N=10	Train		**N=100**	Inner CV folds 1	**N=100**	Inner CV folds 2	**N=100**	Inner CV folds 3	**N=100**	Inner CV folds 4	**N=100**	Inner CV folds 5
N=10	Train		**N=20**	Train_train	**N=20**	Train_train	**N=20**	Train_train	**N=20**	Train_train	**N=20**	Train_validation
N=10	Train		**N=20**	Train_train	**N=20**	Train_train	**N=20**	Train_train	**N=20**	Train_validation	**N=20**	Train_train
N=10	Train	zoom on train =>	**N=20**	Train_train +	**N=20**	Train_train +	**N=20**	Train_valida +	**N=20**	Train_train +	**N=20**	Train_train
N=10	Train		**N=20**	Train_train	**N=20**	Train_validation	**N=20**	Train_train	**N=20**	Train_train	**N=20**	Train_train
N=10	Train		**N=20**	Train_validation	**N=20**	Train_train	**N=20**	Train_train	**N=20**	Train_train	**N=20**	Train_train
N=10	Train											
N=10	Train											

Outer Cross Validation: is used to generate a prediction on each sample as a testing set, that is using a model that was neither trained nor validated on this sample before. For each of the 10 outer folds from 1 to 10 (not the zero one!), train and tune a model using the inner cross validation on the remaining 9 folds. Then use this model to predict the target on the testing fold. In the end, assemble the prediction of each testing fold to get a testing prediction for every sample of the dataset.

Inner Cross Validation: is used to find the best hyperparameter(s) during the tuning for each specific fold of the outer cross validation. For example for the 1st fold of the outer cross validation, we are left with 90 samples used to train and validate the models. These 90 samples can be divided in 5 folds to perform an inner cross validation to tune the model that will then be used to predict the value on the 10 samples of the testing set fold.

Fold 0: Fold 0 is used to test a different hypothesis. Here, we are not investigating R2=f(Age), but Coef/Relative importance of variable V = f(Age). Do do that, all we need to do in to tune a model. We don't necessarily need to test its performance. So we do not need a test set, only a validation set. So we can do a single, *not nested* cross validation (the inner one). Because we have no test set, we can leverage the testing data and feed them as training/validation data instead. Hence the slightly bigger sample sizes (100 instead of 80; 20 instead of 18).

Comparison between Nested-Cross validation and other validation methods: 1-Split train/test. Split train/test works when you don't have hyperparameters to tune. Otherwise you're going to overfit when you test on the same samples you tuned the hyperparameters. 2-Because of that people who need to tune hyperparameters usually use a train/validation/test split. They train on train, tune the hyperparameters on validation, and evaluate the unbiased performance of model on the untouched test dataset. 3-CV(train)/test. With 2 (double split train/validation/test), the tuning of the hyperparameter is not leveraging the whole data as much as with a cross validation. So people ususally replace of the two splits, the training/validation split, by a cross validation instead. But they keep a split between train+validation and test. This is probably the most common setting. 4-Nested cross validation. To fully leverage the whole dataset, nested cross validation replaces both splits by a cross validation. So not only the train/validation split, but also the train+validation/test split. That's why it's nested.
