# Supplementary material for: A systematic machine learning and data type comparison yields metagenomic predictors of infant age, sex, breastfeeding, antibiotic usage, country of origin, and delivery type
Source: PLoS Comput Biol. 2020 May 11;16(5):e1007895. doi: 10.1371/journal.pcbi.1007895 (PMC7241849; doi:10.1371/journal.pcbi.1007895)
Supplement: S5 Fig — (PDF) [file pcbi.1007895.s015.pdf]

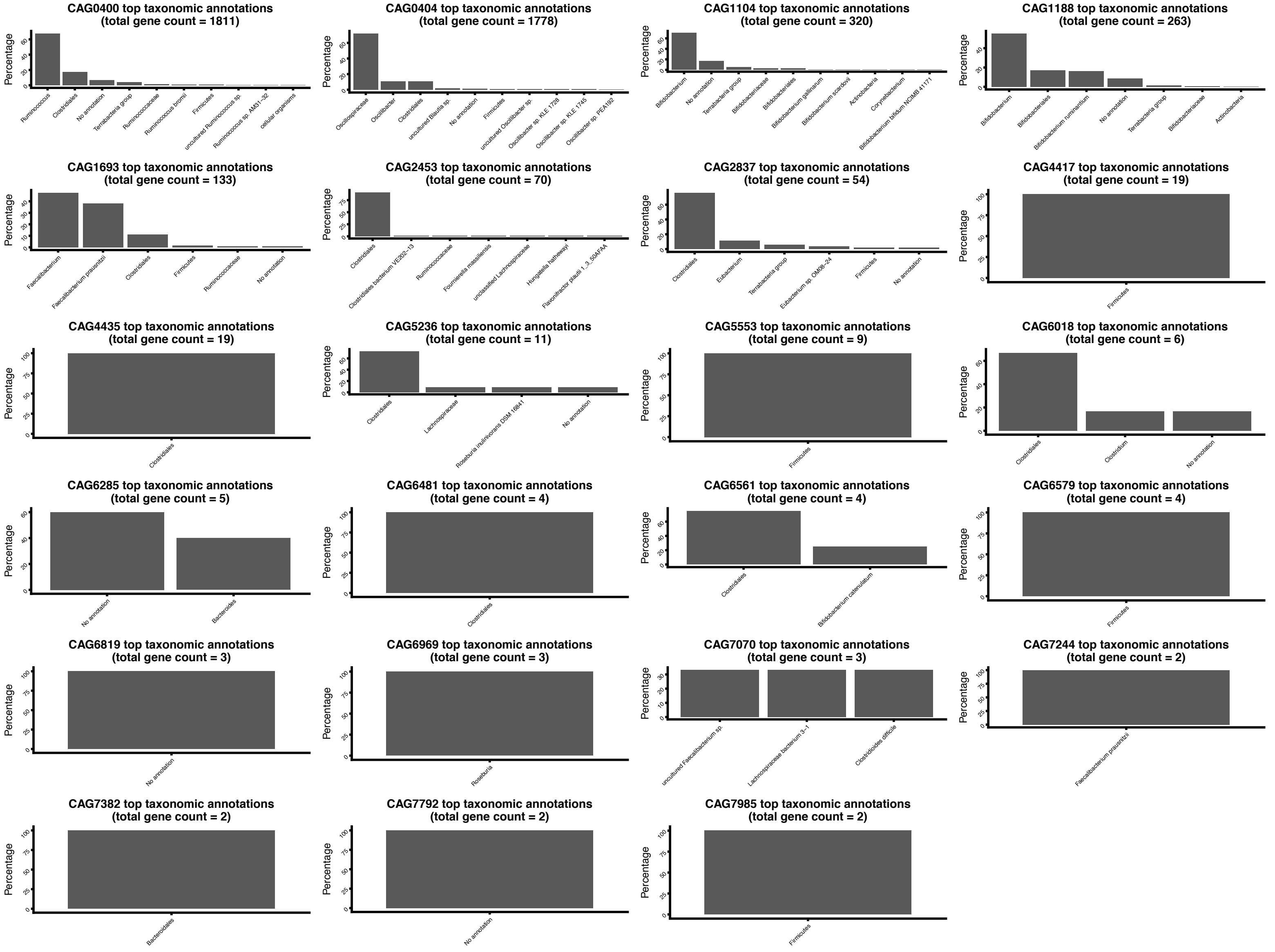
CAG0400 top taxonomic annotations
(total gene count = 1811)
Percentage
Ruminococcus
Clostridiales
No annotation
Terrabacteria group
Ruminococcaceae
Ruminococcus bromii
Firmicutes
uncultured Ruminococcus sp.
Ruminococcus sp. AM31−32
cellular organisms
CAG0404 top taxonomic annotations
(total gene count = 1778)
Oscillospiraceae
Oscillibacter
Clostridiales
uncultured Blautia sp.
No annotation
Firmicutes
uncultured Oscillibacter sp.
Oscillibacter sp. KLE 1728
Oscillibacter sp. KLE 1745
Oscillibacter sp. PEA192
CAG1104 top taxonomic annotations
(total gene count = 320)
Bifidobacterium
No annotation
Terrabacteria group
Bifidobacteriaceae
Bifidobacteriales
Bifidobacterium gallinarum
Bifidobacterium scardovii
Actinobacteria
Corynebacterium
Bifidobacterium bifidum NCIMB 41171
CAG1188 top taxonomic annotations
(total gene count = 263)
Bifidobacterium
Bifidobacteriales
Bifidobacterium ruminantium
No annotation
Terrabacteria group
Bifidobacteriaceae
Actinobacteria
CAG1693 top taxonomic annotations
(total gene count = 133)
Faecalibacterium
Faecalibacterium prausnitzii
Clostridiales
Firmicutes
Ruminococcaceae
No annotation
CAG2453 top taxonomic annotations
(total gene count = 70)
Clostridiales
Clostridiales bacterium VE202−13
Ruminococcaceae
Fournierella massiliensis
unclassified Lachnospiraceae
Hungatella hathewayi
Flavonifractor plautii 1_3_50AFAA
CAG2837 top taxonomic annotations
(total gene count = 54)
Clostridiales
Eubacterium
Terrabacteria group
Eubacterium sp. OM08−24
Firmicutes
No annotation
CAG4417 top taxonomic annotations
(total gene count = 19)
Firmicutes
CAG4435 top taxonomic annotations
(total gene count = 19)
Clostridiales
CAG5236 top taxonomic annotations
(total gene count = 11)
Clostridiales
Lachnospiraceae
Roseburia inulinivorans DSM 16841
No annotation
CAG5553 top taxonomic annotations
(total gene count = 9)
Firmicutes
CAG6018 top taxonomic annotations
(total gene count = 6)
Clostridiales
Clostridium
No annotation
CAG6285 top taxonomic annotations
(total gene count = 5)
No annotation
Bacteroides
CAG6481 top taxonomic annotations
(total gene count = 4)
Clostridiales
CAG6561 top taxonomic annotations
(total gene count = 4)
Clostridiales
Bifidobacterium catenulatum
CAG6579 top taxonomic annotations
(total gene count = 4)
Firmicutes
CAG6819 top taxonomic annotations
(total gene count = 3)
No annotation
CAG6969 top taxonomic annotations
(total gene count = 3)
Roseburia
CAG7070 top taxonomic annotations
(total gene count = 3)
uncultured Faecalibacterium sp.
Lachnospiraceae bacterium 3−1
Clostridioides difficile
CAG7244 top taxonomic annotations
(total gene count = 2)
Faecalibacterium prausnitzii
CAG7382 top taxonomic annotations
(total gene count = 2)
Bacteroidales
CAG7792 top taxonomic annotations
(total gene count = 2)
No annotation
CAG7985 top taxonomic annotations
(total gene count = 2)
Firmicutes
