# Supplementary material for: A systematic machine learning and data type comparison yields metagenomic predictors of infant age, sex, breastfeeding, antibiotic usage, country of origin, and delivery type
Source: PLoS Comput Biol. 2020 May 11;16(5):e1007895. doi: 10.1371/journal.pcbi.1007895 (PMC7241849; doi:10.1371/journal.pcbi.1007895)

Correlation of CAG abundance with age, 3ct cohort

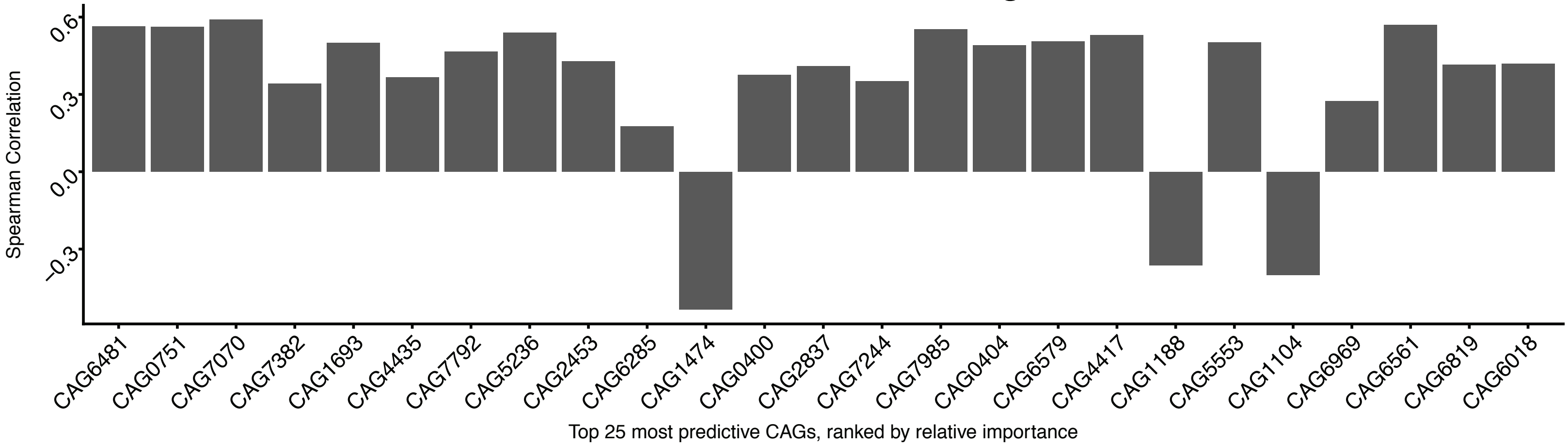

Correlation of CAG abundance with age, abx cohort

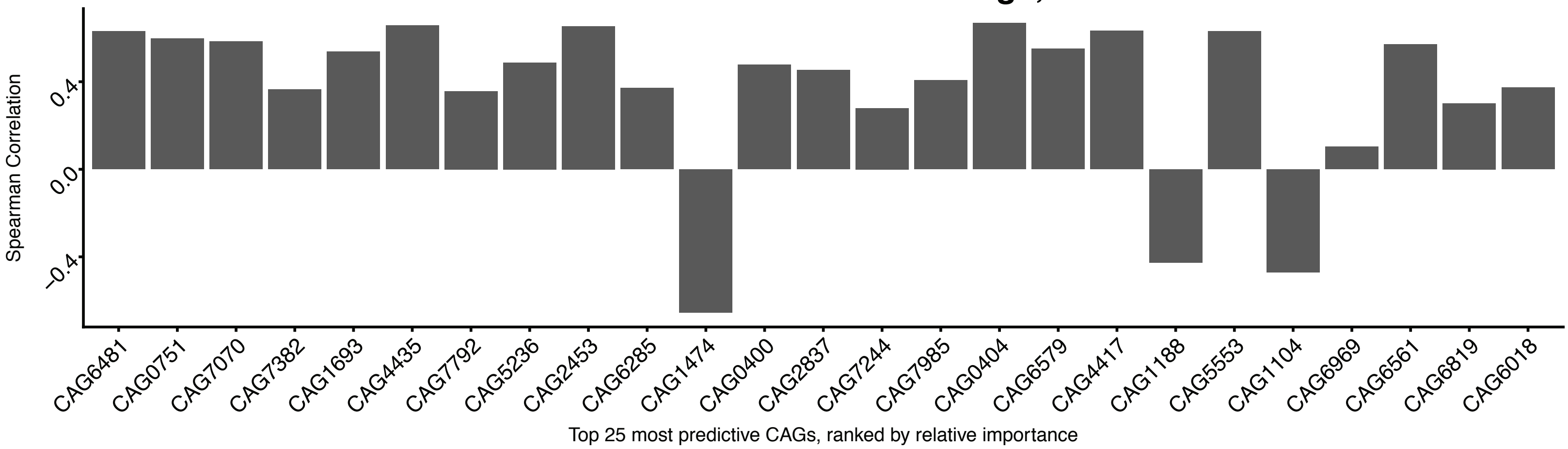

Correlation of CAG abundance with age, bgi cohort

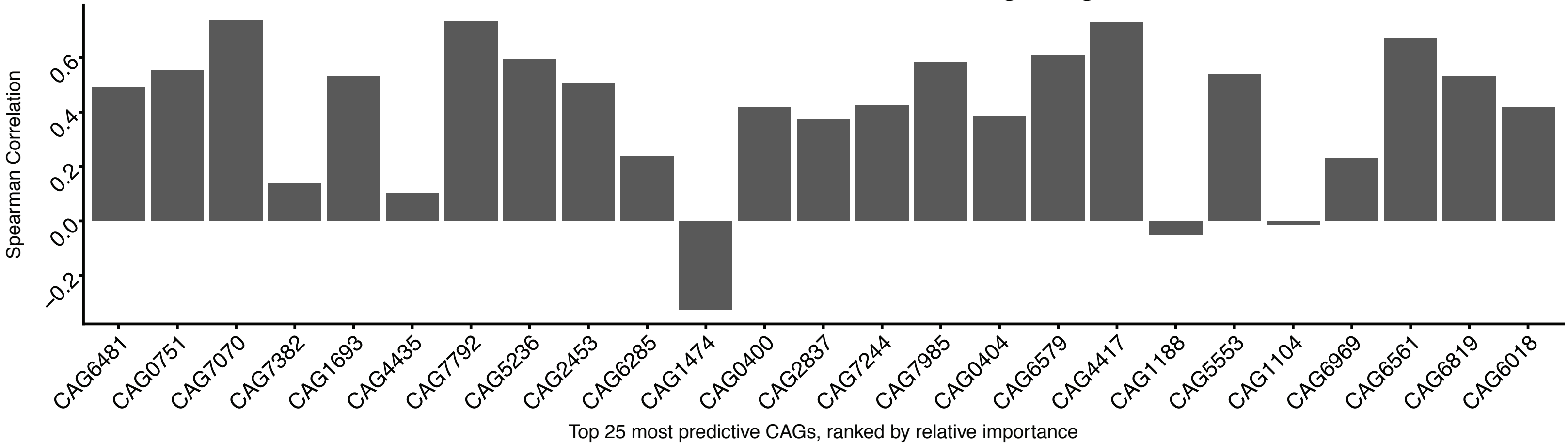

Correlation of CAG abundance with age, t1d cohort

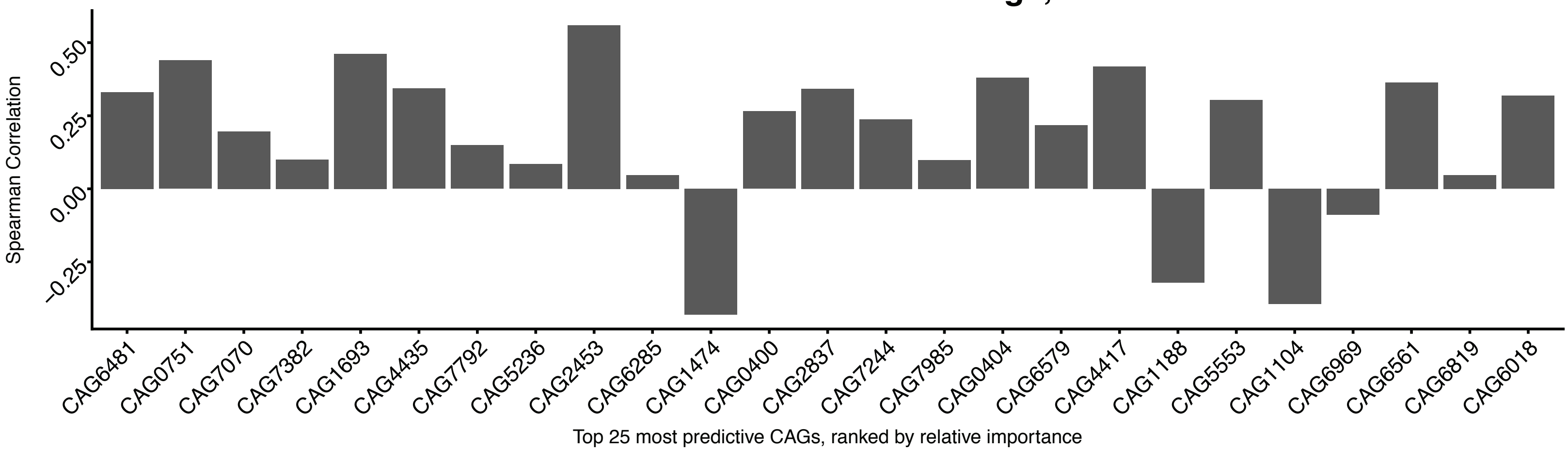

Supplement: S7 Fig — (PDF) [file pcbi.1007895.s017.pdf]
