# Supplementary material for: A systematic machine learning and data type comparison yields metagenomic predictors of infant age, sex, breastfeeding, antibiotic usage, country of origin, and delivery type
Source: PLoS Comput Biol. 2020 May 11;16(5):e1007895. doi: 10.1371/journal.pcbi.1007895 (PMC7241849; doi:10.1371/journal.pcbi.1007895)
Supplement: Attachment — Submitted filename: plos_comp_bio_review.pdf [file pcbi.1007895.s019.pdf]

Alan Le Goallec and Braden T Tierney et al. compared the performance of different machine learning algorithm using varying implementations trained on a data set compiled of four different individual studies across four different types of metagenomic data. They find a strong dependency of both data type and machine learning algorithm on the performance of the classifiers. The training and evaluation of the classifiers is thorough and well described. They obtain good performance for predicting general antibiotic usage, country of origin and breast feed status and delivery type. Notably, models of antibiotic usage and breast feed status only marginally exceeded classification performance of a baseline model trained on non-metagenomic demographics data. The performance estimates for sex prediction are considerably lower.

The study Le Goallec and Tierney et al. conducted addresses important issues of the application of machine learning in metagenomics but I think the authors run into some problems with their approach: The performance estimates in Table 1 indicate that some demographic variables are highly predictive for two out of three phenotypes and add predictive power for five out of six phenotypes. The authors have clearly identified a major confounder of prediction performance. I would like to ask them to provide more details on the demographic variables used in the baseline model and on which demographic variables specifically turned out to be predictive. I think there is a risk that these demographic variables could be quite different across the four different studies and that therefore the demographics is correlated with the study membership of a sample. Consequently, this raises the questions whether the signal they pick up with solely metagenomic based classifiers could partly be the sample study membership leaking through the demographics into the metagenomic data.
I appreciate that the authors ensure that in their cross-validation approach samples from the same individual were attributed to different folds. In a similar fashion they should also consider avoid assigning individuals in the same study to both the training and testing folds at the same time so that they can get an unbiased estimates. They could also look into cross-cohort validation as Pasolini et al. did in https://journals.plos.org/ploscompbiol/article?id=10.1371/journal.pcbi.1004977. Similarly, the authors could try to partial out demographics or study membership from the training data and re-train the classifiers on the transformed data.
Finally, I would very much like to see some exploratory analysis of the covariates analyzed in this study. I think there would be some room for further figures at the beginning of the results to describe the data set. For example, is there any co-clustering of the study membership with any of the host phenotypes based on e.g. a principal component analysis?
Could the authors please also motivate why they picked these four studies? Why did they choose a data set with host phenotypes such as sex and age over a data set with e.g. clinically more relevant disease phenotypes? Were the different phenotypes available for all samples?

The authors write “To our knowledge, we are the first to successfully predict infant age and sex from microbiome data” - I think a AUC of 0.605 for sex prediction is not overly successful. An R^2 of 0.625 is hard to judge without knowing the distribution of age across people. How much off is the age prediction in units of time? This would be helpful to interpret the estimate of the

R^2 the authors provide. It would also be great to know the sensitivity and specificity they obtained throughout the classification tasks. Along the same lines, I find the title slightly misleading as talking of a robust prediction implies that the performance is consistently quite high. Also, it's not only the metagenomic data that is used in the prediction but also the demographics.

Could the authors also report whether there was any class imbalance in their data set? If that is the case, I would suggest to include precision/recall curves along side the receiver operating characteristics, which can be skewed when the class label distribution is imbalanced.

I was also struggling to understand the concordance analysis. Maybe presenting the results in an additional figure would help the reader to understand this part better. The authors also write "We chose not to investigate genes as each phenotype was not analyzed with the same set of genes" I didn't quite understand why the phenotypes were not analyzed with the same set of genes.

I noted that Sup Table 1 is not a table but rather a schematic of the nested cross validation. I suggest to convert this table into a figure instead.

"We removed samples where one of the CAGs was missing" Could the authors explain how a CAG can be missing?

The authors report several CAGs of a few hundred genes as markers for ageing. A typical bacterial species has got more genes. Could the authors provide some statistics for the number of genes per CAG that they obtained?

The authors perform a feature selection step by selecting features which were significantly correlated with the phenotype in the classification. I wonder if the authors miss any signal this way? A feature could improve phenotype prediction in combination with other features but not be directly associated with the phenotype. Could the authors report how many associated taxa, CAGs and pathways there were per phenotype?

It's great that the authors make their code available on GitHub and provide additional results on a web site. Unfortunately I wasn't able to access the web site trying different browser over several days.

I didn't quite understand how the authors derived their gene-level features. In the Methods section 'Identification of genes for testing' they state that they get the genes for their 'gene-level' analysis from CAGs that they extracted from the Caret's Gradient Boosted Machine classifiers. Did they use these genes as input features for training the classification algorithms? In other words are they using the phenotype labels twice? Once for CAG extraction and once for gene-level machine learning classifier training?

The authors are using a metagenomic binning approach on a gene-level basis to derive CAGs. It might be worth investigating alternative approaches. Other recent binning methods such as MaxBin 2.0 https://academic.oup.com/bioinformatics/article/32/4/605/1744462 (also see https://www.nature.com/articles/s41579-018-0029-9) not only use co-abundance information but also features of sequence composition and operate directly on contigs. This way they also keep genomic neighborhood information. Mapping genome bins to the taxonomy to interpret CAG features would become easier this way.

Related, I was surprised that of the top 100 genes there were 72% hypothetical genes. That seems like a really high number. Did the authors double check the annotation process? Could they map back some genes to the assemblies to rule out that the gene calling or annotation is due to problematic assemblies? A common phenomenon is that genes at the end of contigs are only partially assembled and might not represent proper open reading frames.

I am aware that the authors already provide an extensive array of different ML classifier and data types. However, I think it would be quite insightful to investigate how a reference database-based gene-level approach such as a metagenome-level Pfam annotation or eggNOG annotation would compare to a de novo assembly gene-level approach.

I don’t think including genomic variation as an additional data type is necessary but I think the authors should discuss it as a potential data type i.e. structural variation (also see the second paper the authors cite) and variation at the single nucleotide level.

“In accordance with the literature, we removed short genes ….” Please give a reference.

Pick more divergent colors in the performance plots which are easier to distinguish e.g. using a ColorBrewer palette.

I noticed that there were several performance values that were negative in Sup Tab 2 especially for the ElasticNet approach. I think something must have gone wrong. I also noticed several NA performance values for the SVM. Could the authors please explain how this came about?

I would make reviewing the paper much easier if the authors could provide page and line numbers and include the figures in the text.

The authors write “The exception to this rule was for the Antibiotic Usage phenotype, which primarily had annotated genes, some of which were associated with common pathways of antibiotic resistance, like outer membrane assembly (BamA), DNA repair (XerD), and efflux (YycB).”
Please provide some references here. XerD is a recombinase not involved in DNA repair but rather in integration of genetic material such as resistance genes.
