# Supplementary material for: A systematic machine learning and data type comparison yields metagenomic predictors of infant age, sex, breastfeeding, antibiotic usage, country of origin, and delivery type
Source: PLoS Comput Biol. 2020 May 11;16(5):e1007895. doi: 10.1371/journal.pcbi.1007895 (PMC7241849; doi:10.1371/journal.pcbi.1007895)
Supplement: Attachment — Submitted filename: PLOS_CB_Le_Goallec_and_Tierney_et_al_review.pdf [file pcbi.1007895.s020.pdf]

The authors present an analysis of 1570 microbiome samples from 300 infants from four countries. They appear to have two main goals: 1) to characterize the dataset and 2) to show how analytical choices affect the results of that characterization. They show broad variation in prediction accuracy based on modeling choices, and make several insightful recommendations for how practitioners should proceed. They also present interesting results about co-abundance groups that predict infant age, and some insights on the utility of different metagenomic features for this and other purposes.

In general, I think there should be more papers like this with a rigorous analysis of how modeling choices affect results. I recommend this paper for publication, however I do have some comments that I would like to see addressed.

Major comments:

1) It is concerning that the demographics can be predicted from each other at all. In particular, geography and sex seem tightly correlated. This is a fundamental limitation of the dataset that needs more attention and discussion.
2) Of the non-demographic traits only birth-type has a significant increase in accuracy after adding microbiome to the model. This seems particularly interesting as it has a natural causal direction (birth type -> changes in microbiome). I think more attention should be paid to the results of this model, in the same way as for age. Are there genes/taxa/etc that seem to be affected by birth type? Could these have any biological or medical implications?
3) My intuition is that the lack of significant effects for the other non-demographic phenotypes seems to be driven more by the high predictive power of the demographics than a lack of signal from the microbiome. It would be interesting to see if these results changed on a more homogeneous sub-cohort, where the predictive power of the demographics would be reduced. This could be as simple as separate male/female analyses.
4) I would not say that the authors have “successfully predicted sex from the microbiome” (p9). The vast majority of the predictive power for sex is coming from the fact that it is tightly coupled to geography, and while the additional contribution of the microbiome is statistically significant it is also small. This claim should be closer to “we have identified genes/taxa/etc that are correlated with sex”.
5) To deal with the fact that some individuals contribute multiple samples, the authors enforce the constraint that all samples from an individual are in the same fold. This is fine, but there are other ways to deal with this (choosing one sample per person, learning individual-specific profiles and correcting for them). This is an unexplored aspect of potential variation in the results. Since variation in results due to analytical choices is the main theme of the paper, that choice should at least be highlighted before the method section.

Minor comments:

- Perhaps the ordering of the results should be changed. The abstract, introduction and discussion all heavily focused on the question of predictive consistency, but the results are primarily about the biological implications of the analysis until the last section.
- Author affiliation 6 is missing a department
- The concordance of feature importance between methods seems particularly relevant, perhaps Supp Fig 6 should be a main Figure. However, I can’t seem to access the Supp Figures, did the author’s forget to upload them? I see only supp tables 1-5.
