# Supplementary material for: A systematic machine learning and data type comparison yields metagenomic predictors of infant age, sex, breastfeeding, antibiotic usage, country of origin, and delivery type
Source: PLoS Comput Biol. 2020 May 11;16(5):e1007895. doi: 10.1371/journal.pcbi.1007895 (PMC7241849; doi:10.1371/journal.pcbi.1007895)
Supplement: Attachment — Submitted filename: response_to_reviewers.pdf [file pcbi.1007895.s021.pdf]

2/19/2020

Dear Drs. Papin, Segata, and reviewers:

We thank the editors and reviewers for their consideration of our manuscript and insightful comments. We have sought to integrate them into the manuscript and believe this new draft is much improved.

Here, we both summarize the overall changes and, in the proceeding document, point-by-point responses in blue text to each critique raised, quoting specific changes to the main text. We additionally have uploaded a version of the manuscript main text with changes tracked. We hope the reviewers and Journal will find these changes satisfactory, and we look forward to a favorable response.

The major changes to our manuscript are as follows:

(1) We have clarified the key conclusions from our analyses regarding variation in prediction accuracy due to algorithm and microbiome data type choice.
(2) We have sought to provide further justification and Introduction for our efforts in the discussion.
(3) We have provided specific recommendations for readers considering carrying out a microbiome prediction task.
(4) We have reordered the Results, grouping the machine-learning-specific conclusions regarding model concordance (now a main figure) and output together while similarly grouping the biological association results.
(5) We additionally have reduced the strength of claims regarding our predictive capability for sex and age, modifying the Title and Discussion.
(6) Finally, we carried out the specific analyses and modifications requested by the reviewers, including a discussion of class imbalance, exploratory analysis of our phenotypic data, and justification of our methodological choices.

On behalf of the co-authors,

Chirag Patel and Aleksander Kostic

Reviewer #1: The authors present a systematic comparison of machine learning tools applied to microbiome data.

Overall, this may be interesting, but I'm lacking three major parts:
1. The motivation is not sufficient to explain what we're gaining in this paper.

We thank the reviewer for all of their constructive comments and critique. We have now re-worked the Introduction to clarify the motivation behind our paper. At the end of the first paragraph, we highlight the issue of reproducibility faced by the microbiome field by writing:

“Therefore, the field often faces problems of reproducibility and difficult-to-interpret results driven by increased variation in methods and study design.[10] For example, Forslund et al demonstrated in a meta-analysis of Type 2 Diabetes -- microbiome associations that results were in large part dependent on whether or not a given study adjusted for metformin usage.(Forslund et al. 2015)”

We go on to write at the head of the second paragraph:

“The motivation for the work described here stems from the challenge of generating reproducible predictive models for host phenotype that use, at least in part, microbiome information as input features. Generalizable and robust modeling is essential for the microbiome to achieve clinical diagnostic utility. In observational studies, it is hypothesized that “Most Research Findings are False” (Ioannidis, *PLoS Medicine* 2005). One source of false findings includes variation in study design, such as choice of model. Variation in findings have been described as a “Vibration of Effects,” (VoE) and it has been shown to drastically affect the direction of the relationship between dependent and independent variables.(Patel et al. 2015)...In this work, we quantify vibration of effects due to algorithmic and data type choice.”

Finally, to highlight the broader need of identifying sources of confounding in the microbiome, we end the Introduction by writing:

“Understanding how the microbiome varies in association to features like age and sex will quantitate the degree of potential confounding by demographic variables in other microbiome-association studies.”

2. Very much related, the outcome of this paper is unclear to me. It reads more like a grocery list of models and their results, and which model will find which variable, without any profound conclusion that the reader can take home.

We have sought to condense the desired outcomes of the manuscript into the final two paragraphs of our Introduction while remaining as transparent and comprehensive as possible. The first paragraph is devoted to the outcomes of the paper associated with variation in model and data type choice, and the second describes the biological associations we have identified as a result. While we know that having both association and model “vibration of effect”s results in a single manuscript is a large amount of information, we claim that one informs the other. Knowing how biological associations change (or do not) as a function of model and data type choice is a key takeaway of the manuscript that we currently do not believe is appreciated in the microbiome field. In short, microbiome findings are often a function of your modeling strategy. Also see reviewer 1, point 5 for specific outcomes and conclusions of the manuscript.

3. Personally, I think papers should be accessible to undergraduate students. This paper is not deductive enough. It is difficult to read. It uses terms (and acronyms) without explaining them, and it doesn't tell a story that the reader can follow.

We agree that our original draft was too opaque. To remedy this point, we have substantially rewritten the text and made sure to define every acronym appropriately when it comes up the first time. We have additionally included a supplementary table with acronym and term definitions. We have reproduced it below:

**Term**	**Acronym**	**Description (if relevant)**
Elastic Net	EB	
Random Forest	RF	
Gradient Boosted Machine	GBM	
Support Vector Machine	SVM	
K-Nearest-Neighbors	KNN	
Naïve Bayes	NB	
Microbial feature		A single unit of observation within the human microbiome – for this paper, this term refers to either a species, pathway, co-abundance-group or gene
Data type		A type of microbial feature (e.g. species vs. genes)
Co-Abundance-Groups	CAGs	A group of co-occurring microbial genes
Pathways		A metabolic pathway as classified by BioCyc.
MetaPhlAn2 taxa/species		Microbial species identified by the MetaPhlAn2 algorithm
Demographic feature/phenotype		One of the features we were aiming to classify/predict (e.g. age). These are also the features that we adjusted for in the baseline model when we were not using them as our variable

		to be predicted.
Baseline model		A predictive model/classifier that only uses demographic data and not microbiome data.
Relative/predictor importance		The relevance of a given microbial feature for predicting a given phenotype
Area Under the Curve	AUC	A measure of predictive accuracy for a model – and AUC of 1 means a model exhibited perfect classification between groups, whereas an AUC of 0.5 means a model is doing no better than randomly choosing between classes.

Supplementary Table 1: Table of acronyms and definitions.

We have additionally reordered our results, putting the concordance and classification findings at the head of the paper after the initial results section to better improve our deductive reasoning and the flow of our manuscript. Now, the vibration of effects data is grouped together, as is the biological data.

4. The introduction is lacking some papers that have worked on these problems before, with addressing their successes and limitations. A big portion of the introduction is really results from this paper. As the reader reaches the real results section the motivation is still unclear.

Maybe if such an intro was present, the authors would not have claimed to be the "first to successfully predict infant age and sex from microbiome data". For example, age was predicted in a paper that examined malnutrition from the Gordon lab (Subramanian et al, Nature 2014), and used to find that malnutritioned children had a "younger microbiome age". If the authors meant to say that others may have predicted age, but they are the first to predict both age and sex, then this current text in misleading. Also, again, the motivation of why such a prediction would be useful is greatly missing.

We agree that the initial draft of the introduction was lacking in all of these dimensions. We now include mention of papers that have worked on the problems of predicting our phenotypes of interest before, including the Subramanian paper mentioned by the reviewer.

To this point, we write in the introduction:

“These phenotypes have been studied to varying degrees in the literature, with many studies reporting associations with breastfeeding and delivery type being.(Ho et al. 2018; Lemas et al. 2016; Pannaraj et al. 2017; Mueller et al. 2015; Chu et al. 2017; Neu 2016) Additionally, de la Cuesta-Zuluaga et al showed that alpha diversity varies with sex across age in adults(de la Cuesta-Zuluaga et al. 2019), Subramanian et al and Galkin et al have built a predictors of age in children and adults, respectively(Galkin et al. 2018; Subramanian et al. 2014), and Jernberg et al identified the presence of long term effects of antibiotic usage on the microbiome.(Jernberg et al. 2010) In additional results stemming from our model and data type comparison, we build on this existing body of literature, identifying novel associations with, specifically, age, sex, delivery type, and antibiotic usage.”

Further, we have reduced the strength of our claims of regarding age and sex prediction, no longer claiming to be the "first to successfully predict age and sex from microbiome data". Instead of claiming novelty, we focus on the biological interpretation of the highly predictive features for each phenotype.

Finally, to address the inclusion of results in the Introduction, we have moved the paragraphs in question (the last paragraphs of the Introduction) to the Results. We agree that these data (creation of the gene catalog, cohort statistics, etc) should not have been in the Introduction to begin with.

5. Overall, I think that comparing many tools could be interesting, but current paper does not teach its readers about why some models are better than others, and does not reveal any novel biological insight from this comparison.

We thank the reviewer for making this comment, as it motivated us to further clarify the following underlying message of our work.

The key teaching statement from our manuscript is that when building predictive models from microbiome data, simply selecting one model and one data type, even if they are based on what has been done by prior studies, is not sufficient and multiple approaches need to be tested in parallel.

We additionally highlight the following specific outcomes regarding model and data type selection from our study here:

A) On variation in output as a function of algorithm choice:

   In the results, we write:

   "Even between the baseline (demographics only) data and the experimental (demographics/microbiome combined) data for the same target phenotype, there wasn't a consensus "best" algorithm...Each algorithm, with the exception of the support vector machines, was the best performer for at least one of the variables tested."

   In the discussion, we write:

   "Here, we demonstrate how variation in modeling approach drastically changes prediction accuracy for a range of microbiome-associated phenotypes. This provides a cautionary note in developing new microbiome-host interaction studies, analyzing existing microbiome studies, and finally, accurately interpreting findings from these studies. We show that there is "no free lunch" in algorithmic choice for microbiome prediction tasks, and that a range of algorithms should be tested to gauge robustness."

B) On selecting linear vs. non-linear algorithms:

   In the discussion, we write:

   "For some phenotypes such as age, the information contained in the microbiome seems to be non-linear. The use of non-linear models such as the GBM may greatly improve performance over

linear models such as the elastic net. For other phenotypes, the information encoded in the microbiome seems to be linear, as no significant difference was observed between the elastic nets and the tree based methods. In this situation, the linear methods might be preferred because of their interpretability."

"We did, however, identify some non-linear algorithms that consistently underperformed: SVMs and the KNN approaches."

On biological relevance, we address the following 4 outcomes in the manuscript:

C) No model is inherently "better" than others, but rather your biological conclusions need to be interpreted in light of the models that you choose, as they will be dependent on both data type and model choice.

We address this point in the discussion:

"We reported biological associations for our most robust phenotype-associated findings, including age, delivery type, sex, and antibiotic usage. We note that biological interpretation is going to be dependent on microbiome data type in addition to model choice"

D) *De novo,* gene-level analyses tend to generate higher prediction accuracies than reference-based approaches (the species and pathway abundance data). That said, the species and gene level analyses identified biologically (taxonomically) similar features.

We mention these points in the results:

"While CAGs outperformed MetaPhlan2 in terms of prediction performance (with maximal R^2 values of 0.312 and 0.625, respectively), we found that for their best-performing models, their most age-associated features had overlap in taxonomic classification (Supp. Table 3)."

And also in the discussion:

"Second, we demonstrate that while it is challenging to find an optimal model for all phenotypes and data types or even identify why some models outperform others, gene-level, reference-free data outperform reference-based approaches in prediction of human host phenotype. Reference-based pathway-level features were never, in our analysis, the most effective at predicting phenotype when compared to the other data types."

"...based on the similarity in biological interpretation between our MetaPhlAn2-based and CAG-based predictors, we show that reference-based approaches can certainly at times yield biologically meaningful associations when the ultimate goal is hypothesis generation, not prediction accuracy."

E) As reported in other studies, we observe age-associated succession in the infant gut microbiome:

To this point, we write in the discussion:

“Specifically regarding age, we claim that we have identified portions of a patterned, predictable microbial succession early in life.[21] Notably, this has been demonstrated in the past to be the case for one of our top hits for age association, *Faecalibacterium prausnitzii*.[22]”

F) The need to follow up the potential existence of “antibiotic scarring”:

To this point, we write in the discussion:

“Therefore, we hope that the associations we have identified can be followed in biological assays or further computational validation in future work. On this note, we are additionally interested in following up how historical antibiotic usage is identifiable in patient cohorts. This “antibiotic scarring” could potentially provide valuable insight into the long-term effects of medication usage on the microbiome.”

Reviewer #2:
Alan Le Goallec and Braden T Tierney et al. compared the performance of different machine learning algorithm using varying implementations trained on a data set compiled of four different individual studies across four different types of metagenomic data. They find a strong dependency of both data type and machine learning algorithm on the performance of the classifiers. The training and evaluation of the classifiers is thorough and well described. They obtain good performance for predicting general antibiotic usage, country of origin and breast feed status and delivery type. Notably, models of antibiotic usage and breast feed status only marginally exceeded classification performance of a baseline model trained on non-metagenomic demographics data. The performance estimates for sex prediction are considerably lower.
We thank the reviewer for all of their critique and time spent on the review.

1. The study Le Goallec and Tierney et al. conducted addresses important issues of the application of machine learning in metagenomics but I think the authors run into some problems with their approach: The performance estimates in Table 1 indicate that some demographic variables are highly predictive for two out of three phenotypes and add predictive power for five out of six phenotypes. The authors have clearly identified a major confounder of prediction performance. I would like to ask them to provide more details on the demographic variables used in the baseline model and on which demographic variables specifically turned out to be predictive. I think there is a risk that these demographic variables could be quite different across the four different studies and that therefore the demographics is correlated with the study membership of a sample. Consequently, this raises the questions whether the signal they pick up with solely metagenomic based classifiers could partly be the sample study membership leaking through the demographics into the metagenomic data.

We thank the reviewer for this comment, as drew our attention to the lack of information we provided on demographic data in the original manuscript draft. We have now sought to remedy this weakness by including a series of tables that contain demographic information for each cohort. Our overview table, listed in the paper as Supplementary Table 2, is included below with its legend:

	Vatanen_2016	Yassour_2016	Backhed_2015	Kostic_2015	Overall
**Number of Subjects**					
	211	38	98	19	366
**Sex (# samples)**					
Female	330 (42%)	102 (45%)	107 (55%)	69 (55%)	608 (46%)
Male	445 (58%)	123 (55%)	86 (45%)	55 (45%)	709 (54%)
**Delivery Type (# samples)**					
cesarean	69 (9%)	30 (13%)	29 (15%)	4 (3%)	132 (10%)
vaginal	706 (91%)	195 (87%)	164 (85%)	120 (97%)	1185 (90%)

**Country (# samples)**					
Estonia	186 (24%)	0 (0%)	0 (0%)	21 (17%)	207 (16%)
Finland	283 (37%)	225 (100%)	0 (0%)	103 (83%)	611 (46%)
Russia	306 (39%)	0 (0%)	0 (0%)	0 (00%)	306 (23%)
Sweden	0 (0%)	0 (0%)	193 (100%)	0 (00%)	193 (15%)
**Exclusive Breastfeeding (# samples)**					
No	269 (35%)	217 (96%)	128 (66%)	35 (28%)	649 (49%)
Yes	506 (65%)	8 (04%)	65 (34%)	89 (72%)	668 (51%)
**Ever used antibiotics (# samples)**					
No	286 (37%)	123 (55%)	166 (86%)	56 (45%)	631 (48%)
Yes	489 (63%)	102 (45%)	27 (14%)	68 (55%)	686 (52%)
**Age (days)**					
Mean	466.8065	518.18	244.3679	590.8548	
Median	448	371.36	150	565	
Standard Deviation	259.5519	360.3453	123.3274	261.7377	

Supplementary Table 2: Cohort statistics by demographic variables.

It is referenced in the text in the results as follows:

"We identified limited class imbalance in our phenotypes of interest, with the exception being delivery type (Total cesarean samples = 132, Total vaginal delivery samples = 1,185, Supp Table 2)."

2. I appreciate that the authors ensure that in their cross-validation approach samples from the same individual were attributed to different folds. In a similar fashion they should also consider avoid assigning individuals in the same study to both the training and testing folds at the same time so that they can get an unbiased estimates. They could also look into cross-cohort validation as Pasolini et al. did in https://journals.plos.org/ploscompbiol/article?id=10.1371/journal.pcbi.1004977. Similarly, the authors could try to partial out demographics or study membership from the training data and re-train the classifiers on the transformed data.

We agree with the reviewer that ideally we should have kept individuals from the same study in the same folds. However, because of the limited number of individuals and samples per individuals in some of the studies, imposing this extra constraint would have left us with highly imbalanced folds in terms of sample sizes. For imbalanced classes, some classes would not have been represented in some folds. This is the

reason why we decided that keeping the samples from each participant in the same fold was a fair trade-off.

That being said, we definitely agree that the issue of confounding variables is a big one. This is the very reason why we built "control" models where we used solely demographics variables as predictors. If most of our predictive power came from the demographics variables, which include country of origin, we would not see a significant difference between the predictive accuracy obtained on the "demographics only" model and the models that leverage microbiome information. As observed in Table 1, there was indeed no significant difference for antibiotic usage and for exclusive breastfeeding. On contrary, significant differences were observed for age, delivery type and sex, which shows that the extra predictive accuracy comes from the microbiome variables and cannot be explained by cohort differences.

We specifically clarify this point in the methods:

"We additionally compared the R^2/ROC of the models containing microbiome data to a "baseline" model, which only contained the demographic information not being predicted (age, sex, or country of origin. These "control" models were meant to determine if most of our predictive power came from the demographics variables. If this were the case, we would not see a significant difference between the predictive accuracy obtained on the "demographics only" model and the models that leverage microbiome information."

And in the results:

"As observed in Table 1, there was indeed no significant difference between the baseline and microbiome-including models for antibiotic usage and for exclusive breastfeeding. On contrary, significant differences were observed for age, delivery type and sex, which shows that the extra predictive accuracy comes from the microbiome variables and cannot be explained by cohort differences. "

4. Finally, I would very much like to see some exploratory analysis of the covariates analyzed in this study. I think there would be some room for further figures at the beginning of the results to describe the data set. For example, is there any co-clustering of the study membership with any of the host phenotypes based on e.g. a principal component analysis?

We thank the reviewer for pointing out the need for exploratory analysis of our demographic variables of interest. Indeed, we saw the same need when preparing the manuscript and included the requested information as an extension of our results on our web resource on the "Associations between variables" tab (see upper tab of the UI). We demonstrate in these an association between pairs of variables (e.g. age and sex) with p-values describing the results of t-test, ANOVA, or Chi-Squared test (depending on the nature of the variables in question) between them. We include the option to select different demographic variables, but all of the microbiome features as well (e.g. you can explore the relationship between a specific CAG and demographic feature). We chose to go this route because we felt, given the importance of exploring demographic features and the scope of this project, we could not reduce the analysis to a small number of plots to be placed the head of the paper. Of course, given the reviewer could not access the website, this work could not be observed, and we apologize for this oversight.

Therefore, in addition to confirming that the web resource functions, we now both describe the resource itself in greater detail in the results, writing:

“Our complete results, as well as a full exploration of the relationships between our demographic variables and cohorts, can be viewed at http://apps.chiragjpgroup.org/ubiome_predictions/. This resource includes the associations between and distributions of features used in training (e.g. age and sex), top microbiome predictors of phenotypes, outcomes for specific models and datatypes, and detailed model training/output information (e.g. sensitivities, specificities, and model hyperparameters).”

For the benefit of the reviewers, we are also including here a few example plots to demonstrate our capability to dynamically explore these data:

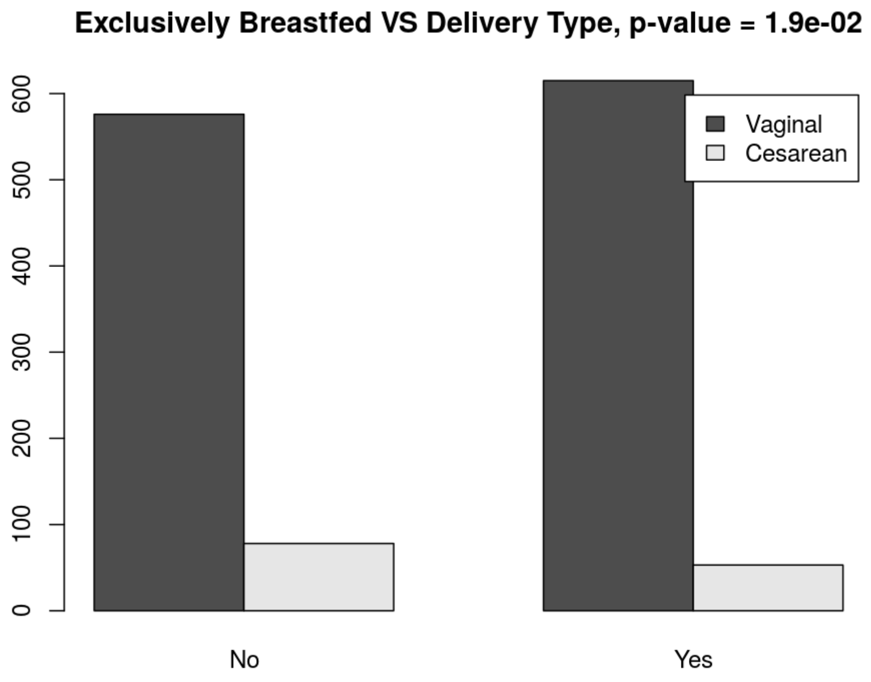

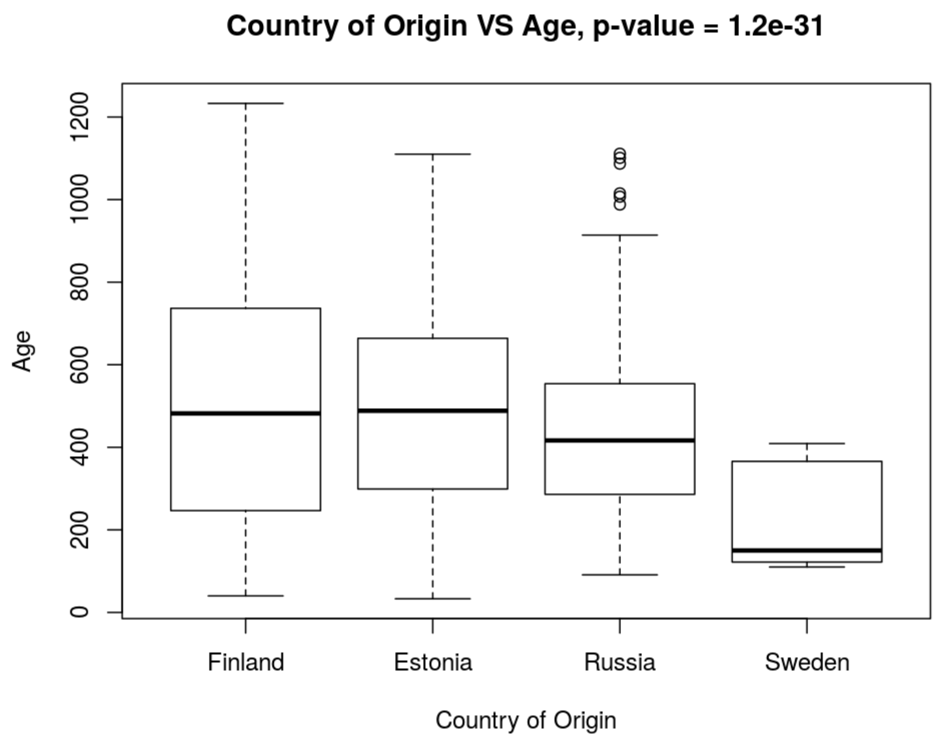

As requested, we additionally have computed a principal component analysis across all of our demographic variables and colored the output by cohort. We now include it as Supplementary Figure 1, and it is reproduced here with its legend:

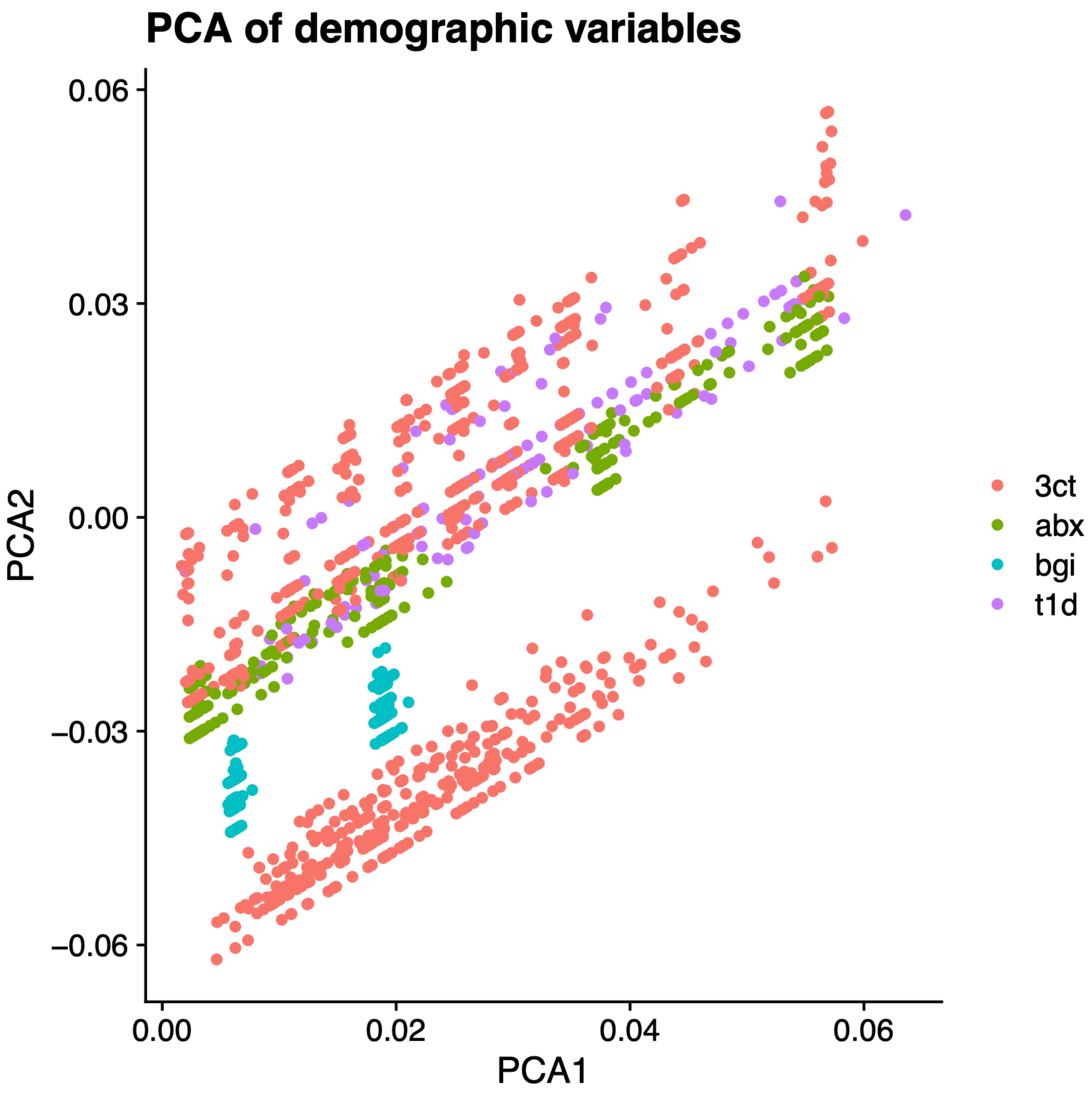

Supplementary Figure 1: Exploratory principal component analysis of all demographic variables, colored by dataset.

We reference this figure in the results by writing

"Preliminary principal component analysis identified minimal stratification between cohorts on the basis of metadata alone (Supp Fig 1)."

5. Could the authors please also motivate why they picked these four studies? Why did they choose a data set with host phenotypes such as sex and age over a data set with e.g. clinically more relevant disease phenotypes? Were the different phenotypes available for all samples?

We selected these four studies because, at the time of writing, they were 1) longitudinal infant microbiome shotgun sequencing studies 2) containing greater than 100 samples 3) and containing overlap on demographic metadata. We found that studies with more clinically relevant phenotypes, while potentially informative, would decrease our overall sample size/power (as they tended to contain fewer patients, and

we only used demographic variables shared across all studies). That being said, certainly future directions for this work outside the scope of this manuscript would unquestionably require investigating greater numbers of clinically relevant data.

We now clarify in the introduction why we selected these four studies, writing:

“We selected these datasets due to their 1) longitudinal nature 2) similar and high-quality metadata and 3) cohort size (>50 samples). Preliminary principal component analysis identified minimal stratification between cohorts on the basis of metadata alone (Supp Fig 1).”

We additionally clarify in the introduction that the demographic variables we used in our models were the across all four cohorts, writing:

“...relative capability of demographic data alone and the microbiome to predict six demographic features (which we also refer to as phenotypes): infant age, sex, breastfeeding status, prior antibiotic usage, delivery type, and home country.”

6. The authors write “To our knowledge, we are the first to successfully predict infant age and sex from microbiome data” - I think a AUC of 0.605 for sex prediction is not overly successful.

We thank the reviewer for this comment and have since removed this statement entirely from the paper. We additionally mention other manuscripts that have worked to predict sex in the past in the introduction, and we focus less on the predictive capability of sex and more on the fact that we have identified a number of robust associations.

7. An R^2 of 0.625 is hard to judge without knowing the distribution of age across people. How much off is the age prediction in units of time? This would be helpful to interpret the estimate of the R^2 the authors provide. It would also be great to know the sensitivity and specificity they obtained throughout the classification tasks.

We thank the reviewer for pointing out this way to increase the interpretability of our results. We computed the root mean squared error (RMSE) for age, and found the R^2 of 0.625+-0.21 is equivalent to a RMSE of 171.0+-5.7 days.

We reference this finding in the results by writing:

“...best improvement in R^2 relative to its demographic baseline (R^2 experimental = 0.625+-0.021, R^2 baseline = 0.113+0-.014, RMSE = 171.0+-5.7 days)”

Additionally, the sensitivity and the specificity obtained throughout the classification tasks can be found on the interactive website under the “Prediction Performances” tab, along other classification metrics. We apologize that the reviewer did not have access to these data beforehand due to issues with the website itself – we have confirmed these are now resolved and that the website can be accessed by a range of browsers in a number of countries (specifically, we tested the ability of individuals to access the site from the UK, Australia, Israel, and multiple states within the US).

8. Along the same lines, I find the title slightly misleading as talking of a robust prediction implies that the performance is consistently quite high. Also, it’s not only the metagenomic data that is used in the prediction but also the demographics.

We have modified the title so that it better reflects the content of the paper. It now reads:

Stress-testing the reproducibility of host-microbiome associations via a systematic machine learning algorithm and data type comparison.

9. Could the authors also report whether there was any class imbalance in their data set? If that is the case, I would suggest to include precision/recall curves along side the receiver operating characteristics, which can be skewed when the class label distribution is imbalanced.

We have added a table (Supp Table 2, reproduced in response to point 1) that recapitulates the class imbalances for the different datasets. Class imbalance was moderate. While we agree that using PR-ROC is a fair way to assess a model's performance accuracy when class imbalance is involved, we have used a different metric for this purpose in this paper: Mean Class Accuracy (MCA, available on the website). The Mean Class Accuracy is calculated as the mean of the accuracy obtained in each class. This way, if a model were to systematically predict the most prevalent class for example, its MCA would be 1/N_classes. Here we obtained significantly higher MCAs.

10. I was also struggling to understand the concordance analysis. Maybe presenting the results in an additional figure would help the reader to understand this part better.

We thank the reviewer for pointing out this lack of clarity in the text. Our "concordance" analysis took place in two parts, which we have now made clarified:

1) The concordance between the ranking of phenotype-associated features across 1) algorithms and 2) data types. In other words, we aimed to determine if algorithms were ranking features similarly in terms of their association with a given phenotype (e.g. Do random forests and elastic nets prioritize the same microbial species in their association with age?). Analogously, we compared the if, for a given phenotype, if Metaphlan2, pathway, or CAG associations were more consistent in terms of relative importance across all algorithms.
2) The similarity between the taxonomic annotation of *de novo* microbiome features and reference-based (Metaphlan2) features in the context of age prediction. We found that, while prediction accuracies varied, the taxonomic annotations of highly age-associated features for both microbiome data types were similar. We claim this indicates the differing use-cases for reference-based or *de novo* approaches – if predicting a phenotype, *de novo* genes may be useful. If looking for associations to motivate biological hypotheses, though, reference-based approaches may be sufficient.

To clarify this analysis, we have done the following:

1) We now describe 1) as "concordance" analysis and 2) as "biological similarity" analysis in order to better distinguish them to the reader.
2) Added the following main figure (reproduced below with its legend) with panels describing concordance analysis described in 1) above.
3) Re-ordered the results such that that the concordance analysis is no longer split up by different sections. The concordance analysis is now listed towards the beginning of the results with the machine-learning-focused (non-biological) results, whereas the biological similarity analysis is towards the end.

We have also sought to clarify our concordance analysis by writing our above comments in the methods:

“We aimed to compute the concordance between the ranking of phenotype-associated features across 1) algorithms and 2) data types. In other words, we aimed to determine if algorithms were ranking features similarly in terms of their association with a given phenotype (e.g. Do random forests and elastic nets prioritize the same microbial species in their association with age?). Analogously, we compared the if, for a given phenotype, if Metaphlan2, pathway, or CAG associations were more consistent in terms of relative importance across all algorithms.”

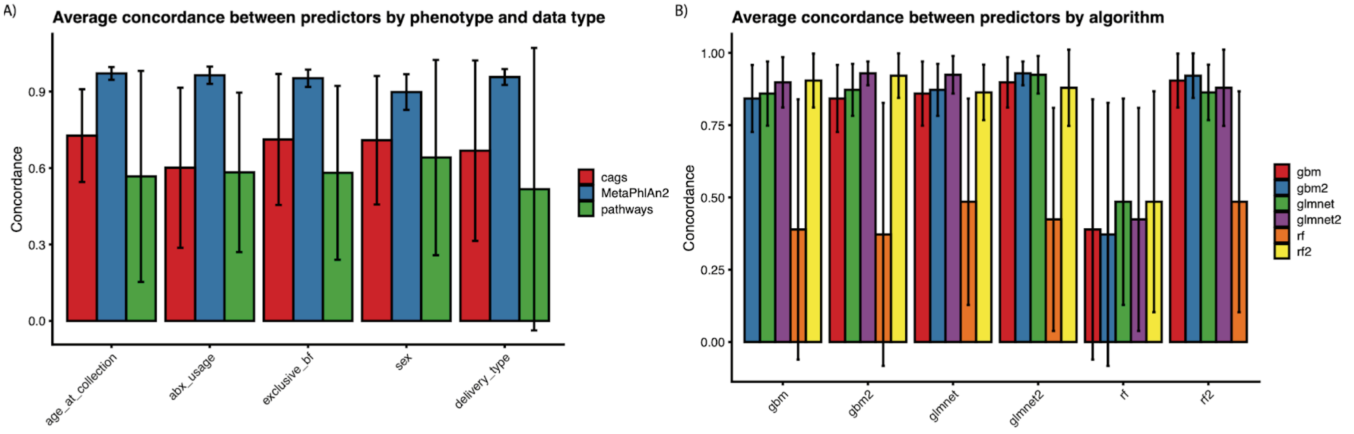

Figure 2: Predictor concordance, measured by similarity in ranking and relative importance, between algorithm, data type, and phenotype.

11. The authors also write “We chose not to investigate genes as each phenotype was not analyzed with the same set of genes” I didn’t quite understand why the phenotypes were not analyzed with the same set of genes.

We agree that this method was not clearly described before. As described in point 16 below, the associations between single genes and phenotype that we tested did not use the same pool of genes for each phenotype. This was because of the computational challenge associated with modeling repeatedly the enormous number of genes present in our combined dataset (10 million). Testing each one was not computationally feasible. Rather, for our gene-based analysis, we used the extracted genes from the top ten most predictive CAGs for every algorithm. This meant that the CAGs, and therefore genes, selected for any two algorithms (e.g.,say an elastic net and a gradient-boosted-machine), would not necessarily be the same. This meant that concordance analysis in the context of the gene-associations would not make sense, as non-overlapping sets of genes were used to predict each phenotype.

To reduce confusion on this point, we have removed the sentence in question from the results section and instead further clarified this point in the methods as follows:

“We first identified CAGs that were identified as highly phenotype-associated for our top performing algorithm for that phenotype. We extracted the individual abundances genes from the top ten most predictive CAGs and then tested the capability of that gene set to predict host phenotype. We did this for two reasons 1) it prevented us from having to test the 10 million genes in our dataset, which was computationally challenging and 2) it allowed us to determine if the averaging of abundances required to compute CAG abundance reduced predictive signal. We additionally note that this approach is not without

drawbacks: specifically, we are implicitly subsetting our gene sets to genes that are likely to already be associated with disease.”

11. I noted that Sup Table 1 is not a table but rather a schematic of the nested cross validation. I suggest to convert this table into a figure instead.

We have now converted this to Supplementary Figure 3, which is reproduced below with its legend:

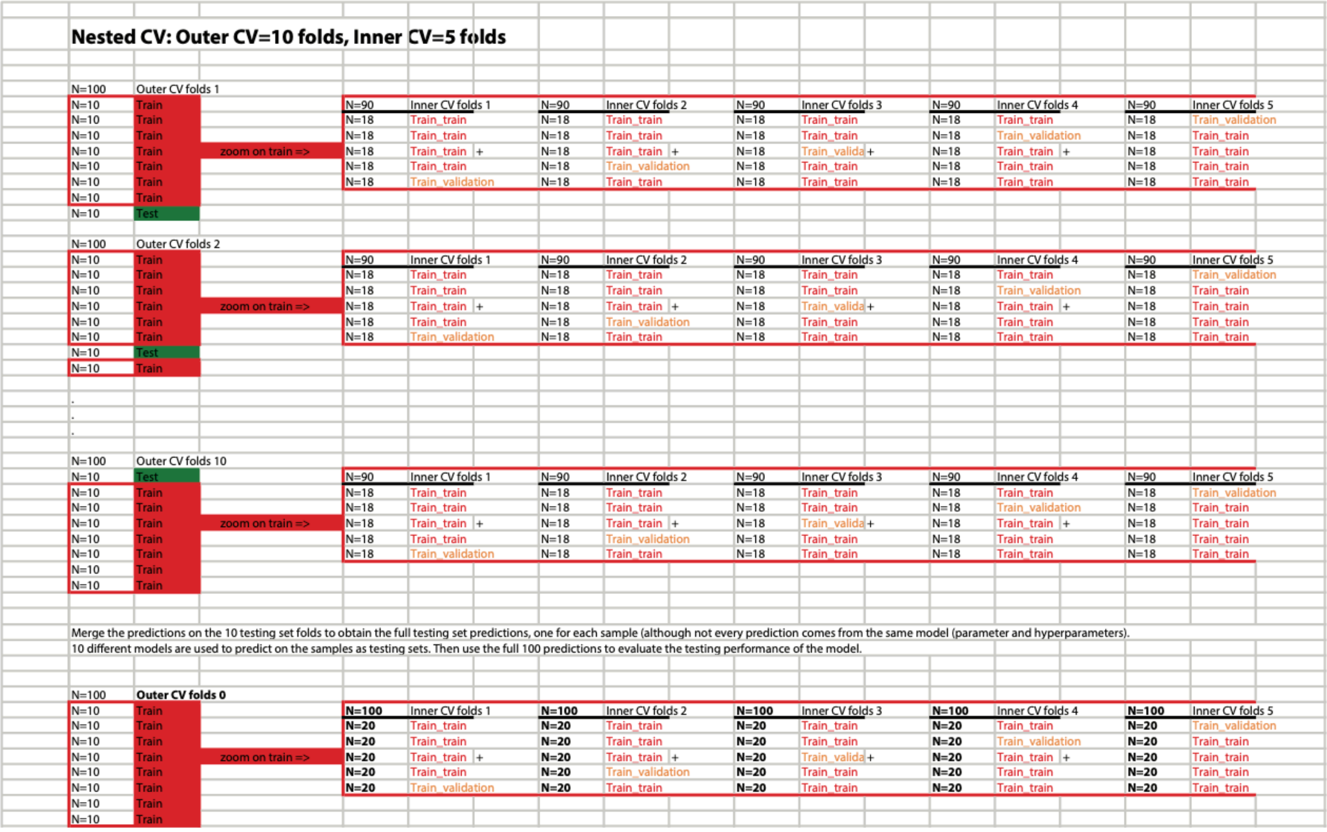

Outer Cross Validation: is used to generate a prediction on each sample as a testing set, that is using a model that was neither trained nor validated on this sample before. For each of the 10 outer folds from 1 to 10 (not the zero one!), train and tune a model using the inner cross validation on the remaining 9 folds. Then use this model to predict the target on the testing fold. In the end, assemble the prediction of each testing fold to get a testing prediction for every sample of the dataset.

Inner Cross Validation: is used to find the best hyperparameter(s) during the tuning for each specific fold of the outer cross validation. For example for the 1st fold of the outer cross validation, we are left with 90 samples used to train and validate the models. These 90 samples can be divided in 5 folds to perform an inner cross validation to tune the model that will then be used to predict the value on the 10 samples of the testing set fold.

Fold 0: Fold 0 is used to test a different hypothesis. Here, we are not investigating R2=f(Age), but Coef/Relative importance of variable V = f(Age). Do do that, all we need to do in to tune a model. We don't necessarily need to test its performance. So we do not need a test set, only a validation set. So we can do a single, *not nested* cross validation (the inner one). Because we have no test set, we can leverage the testing data and feed them as training/validation data instead. Hence the slightly bigger sample sizes (100 instead of 80; 20 instead of 18).

Comparison between Nested-Cross validation and other validation methods: 1-Split train/test. Split train/test works when you don't have hyperparameters to tune. Otherwise you're going to overfit when you test on the same samples you tuned the hyperparameters. 2-Because of that people who need to tune hyperparameters usually use a train/validation/test split. They train on train, tune the hyperparameters on validation, and evaluate the unbiased performance of model on the untouched test dataset. 3-CV(train)/test. With 2 (double split train/validation/test), the tuning of the hyperparameter is not leveraging the whole data as much as with a cross validation. So people ususally replace of the two splits, the training/validation split, by a cross validation instead. But they keep a split between train+validation and test. This is probably the most common setting. 4-Nested cross validation. To fully leverage the whole dataset, nested cross validation replaces both splits by a cross validation. So not only the train/validation split, but also the train+validation/test split. That's why it's nested.

Supplementary Figure 3: Detailed walkthrough and example of our 10x cross-fold validation strategy.

12. “We removed samples where one of the CAGs was missing” Could the authors explain how a CAG can be missing?

We have addressed this lack of clarity. For some samples, assembly failed – we therefore were not able to produce CAGs for this small subset. As such, we removed these samples for our CAG analysis. We have now addressed this in the methods by writing:

“To obtain a complete matrix, we removed the samples for which one of the phenotypes was missing or where assembly failed (therefore resulting in no CAG or gene level data for that sample)”

13. The authors report several CAGs of a few hundred genes as markers for ageing. A typical bacterial species has got more genes. Could the authors provide some statistics for the number of genes per CAG that they obtained?

We now provide a summary of these statistics in Supplementary Figure 2. We reproduce this figure below. We additionally point out in the discussion that these gene clusters are likely functionally linked gene clusters or accessory genomes instead of entire genomes:

“We hypothesize that CAGs’ success in predicting age is likely due to *de novo* assembly capturing genes that are not present in reference databases, meaning that, co-occurrence clusters of those genes capture representations of the “true“ gut microbe core genomes, accessory genomes, or functionally linked genes (as many of our CAGs are not the size of complete microbial genomes).”

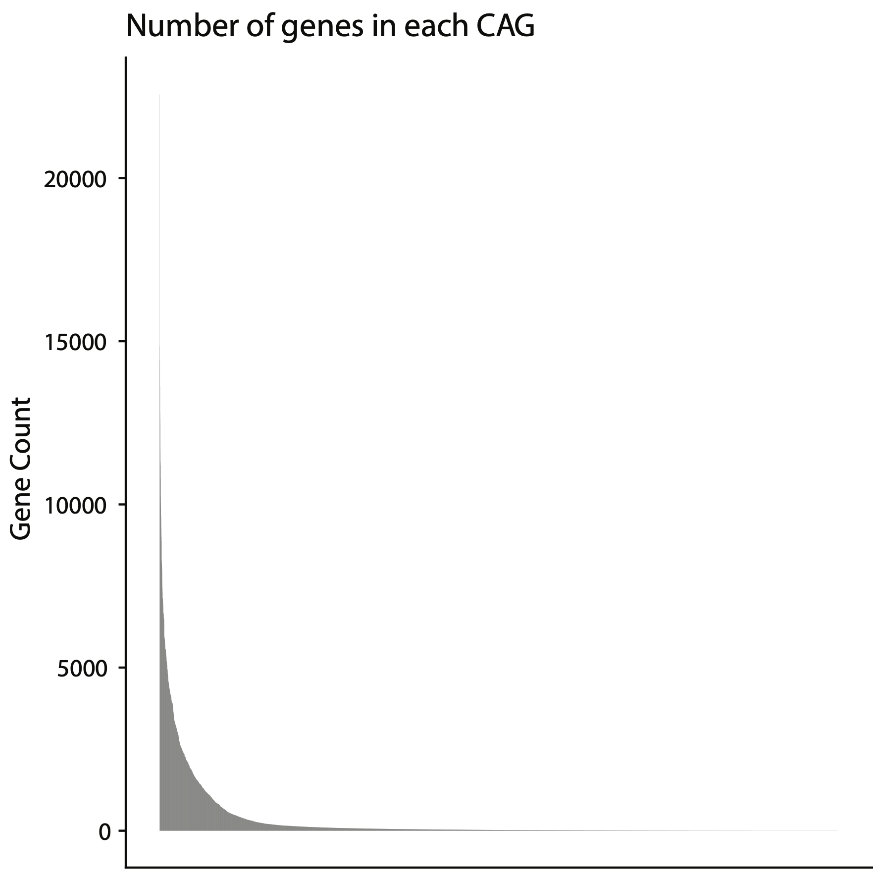

Quantiles

0%	25%	50%	75%	100%
2	5	23	93	22563

Supplementary Figure 2: The number of genes per CAG in our dataset alongside quantiles of the frequency distribution.

14. The authors perform a feature selection step by selecting features which were significantly correlated with the phenotype in the classification. I wonder if the authors miss any signal this way? A feature could improve phenotype prediction in combination with other features but not be directly associated with the phenotype. Could the authors report how many associated taxa, CAGs and pathways there were per phenotype?

It is very likely and plausible that we are missing out on some of the signals because of this first selection step. However, complex models could not handle the very large number of predictors initially at our disposal (up to 20,000), so we had to decide on a method to select a subset of them. It is indeed possible that predictors that were not associated with the phenotype within the context of an univariate model become important within the context of a non-linear multivariate model. However, weakly or uncorrelated univariate predictors are less likely than robust univariate predictors to become robust multivariate predictors. This is the reason why we excluded them in our preprocessing step.

Some algorithms can more easily handle a larger number of predictors (e.g elastic net as opposed to gradient boosted machines) but to compare the performances of the different algorithms, we decided to keep the same number of predictors.

We now report the number of bonferroni-significant associations between the predictors and the phenotype in Supplementary Table 7, and we reproduce the table below and reference it in the methods by saying:

"For each fold, we filtered out the taxa or CAGs or pathways that were not significantly associated with the target variable. For each taxa/CAG/pathway, we ran a linear regression or a logistic regression to predict the phenotype of interest from this unique variable. We report the total associated microbial features in Supplementary Table 7."

Target	Taxa	Pathways	CAGs
Age	677, 37	272, 1010	7669, 623
Sex	714, 0	1278, 4	8292, 0
Country of Origin	584, 130	734, 548	7952, 340
Exclusive Breastfed	348, 366	1281, 1	8290, 2
Antibiotics Usage	714, 0	1099, 183	8275, 17
Delivery type	714, 0	1253, 29	8289, 3

Supplementary Figure 7: Bonferroni corrected associated and statistically significant predictors in a univariate regression/classification context: FALSE, TRUE. The associated features were then fed into our predictive models.

15. It's great that the authors make their code available on GitHub and provide additional results on a web site. Unfortunately I wasn't able to access the web site trying different browser over several days.

We apologize for this inconvenience, especially given how crucial the website results are as extended data in the paper. We have now confirmed across multiple browsers, individuals, and countries (US, UK, Israel, Australia) that the website is indeed functional and accessible.

16. I didn't quite understand how the authors derived their gene-level features. In the Methods section 'Identification of genes for testing' they state that they get the genes for their 'gene-level' analysis from CAGs that they extracted from the Caret's Gradient Boosted Machine classifiers. Did they use these genes as input features for training the classification algorithms? In other words are they using the phenotype labels twice? Once for CAG extraction and once for gene-level machine learning classifier training?

We acknowledge that we used the phenotype labels twice for the genes pipeline: a first time to select the cags from which to extract the genes, and a second time to actually predict the phenotype. We agree that this is non-ideal, as it could lead to a slight overfitting to the testing set.

In a perfect world, we would have extracted a different set of genes for every fold of the outer cross validation, based on which CAGs were selected on this specific fold. However, we do not think this level of preprocessing would lead to substantial bias in the performance of the genes. For each outer cross validation fold, the testing set represents only 10% of the samples so the CAGs extracted would not have been significantly different. The 10 CAGs extracted usually each contain hundreds or thousands of genes, the majority of which are not used by the model. We think the two most significant steps to select which genes are important are the preprocessing step that filters most genes out based on univariate models, and the actual learning on the genes as predictors, both of which do not have access to the testing set.

We have now extended the methods section to clarify, justify, and address the drawbacks of this approach by writing:

"We first identified CAGs that were identified as highly phenotype-associated for our top performing algorithm for that phenotype. We extracted the individual abundances genes from the top ten most predictive CAGs and then tested the capability of that gene set to predict host phenotype. As with the other data types, we filtered genes out that did not have bonferroni-corrected statistically significant univariate associations with phenotypes. We did these filtering steps for two reasons 1) it prevented us from having to test the 10 million genes in our dataset, which was computationally challenging and 2) it allowed us to determine if the averaging of abundances required to compute CAG abundance reduced predictive signal. We additionally note that this approach is not without drawbacks: specifically, we are implicitly subsetting our gene sets to genes that are likely to already be associated with disease."

17. The authors are using a metagenomic binning approach on a gene-level basis to derive CAGs. It might be worth investigating alternative approaches. Other recent binning methods such as MaxBin 2.0 https://academic.oup.com/bioinformatics/article/32/4/605/1744462 (also see

https://www.nature.com/articles/s41579-018-0029-9) not only use co-abundance information but also features of sequence composition and operate directly on contigs. This way they also keep genomic neighborhood information. Mapping genome bins to the taxonomy to interpret CAG features would become easier this way.

We thank the reviewer for this comment and completely agree that using alternative binning approaches to identify biological gene clusters would be useful and quite biologically informative. We believe, however, that for this manuscript given the computational time required to run our pipeline and the goal of our manuscript (comparing the performance of different microbiome data structures and algorithms). If we had a greater focus on taxonomy, certainly using binning methods that take into account contigs would be helpful. In this context, though, we do not believe doing this is within the scope of our work.

Instead of rerunning our pipeline, though, we have discussed alternative data types and algorithmic approaches to their generation in greater depth in the discussion:

"Finally, future expansions on our analysis could include investigating how single nucleotide variation in genes(Costea et al. 2017) – or alternative taxonomic binning approaches(Wu et al. 2016) – function as predictors of host phenotype."

18. Related, I was surprised that of the top 100 genes there were 72% hypothetical genes. That seems like a really high number. Did the authors double check the annotation process? Could they map back some genes to the assemblies to rule out that the gene calling or annotation is due to problematic assemblies? A common phenomenon is that genes at the end of contigs are only partially assembled and might not represent proper open reading frames.

We certainly agree that 72% hypothetical genes is high. This number came from the raw output of Prokka(Seemann 2014), which uses a range of tools (e.g. Pfam,. TIGRFAM) to annotate predicted ORFs. That being said, it is quite possible that *ab initio* annotation (Prokka's approach), could miss certain annotations that annotation by homology (alignment) could catch. Fortunately, our pathway-level analysis used annotation with Biocyc pathways by homology to our gene catalog.

As such, to address the reviewer's comment, we now include the pathway-level BioCyc annotations for each of the gene's of interest in the Table in question (reproduced below). We additionally include the full pathway level annotations for all genes as Supplementary Table 5 (this is too large to reproduce here, but it can be found in our uploaded data).

It is worth noting, however, that annotations by homology alone can cause spurious results, as sequence identify does not always correlate to function. We now state this clearly in the discussion as a potential drawback, writing:

"For example, pathway annotations can be fraught with the challenge of spurious assignment to function due to sequence homology.(Frith 2011)"

19. I am aware that the authors already provide an extensive array of different ML classifier and data types. However, I think it would be quite insightful to investigate how a reference database-based gene-level approach such as a metagenome-level Pfam annotation or eggNOG annotation would compare to a de novo assembly gene-level approach.

We thank the reviewer for this comment. Our pathway comparison is meant to address this point, as we used reference-based annotation by homology with BioCyc Tier 3 to annotate genes. We now explicitly mention it in the results, writing:

“Pathway-level annotations were never the best performing predictors for any phenotype.”

And in the discussion:

“Second, we demonstrate that while it is challenging to find an optimal model for all phenotypes and data types or even identify why some models outperform others, gene-level, reference-free data types outperform reference-based approaches in building microbiome-based predictors. That said, reference-based pathway-level features were never the most effective at predicting phenotype when compared to the other data types.”

20. I don’t think including genomic variation as an additional data type is necessary but I think the authors should discuss it as a potential data type i.e. structural variation (also see the second paper the authors cite) and variation at the single nucleotide level.

We thank the reviewer for this excellent idea. We now reference this point in the discussion, saying:

“Finally, future expansions on our analysis could include investigating how single nucleotide variation in genes(Costea et al. 2017) – or alternative taxonomic binning approaches(Wu et al. 2016) – function as predictors of host phenotype.”

21. “In accordance with the literature, we removed short genes ....” Please give a reference.

We now reference this point with Nielsen et al. 2014.

Nielsen, H. Bjørn, Mathieu Almeida, Agnieszka Sierakowska Juncker, Simon Rasmussen, Junhua Li, Shinichi Sunagawa, Damian R. Plichta, et al. 2014. “Identification and Assembly of Genomes and Genetic Elements in Complex Metagenomic Samples without Using Reference Genomes.” *Nature Biotechnology* 32 (8): 822–28.

22. Pick more divergent colors in the performance plots which are easier to distinguish e.g. using a ColorBrewer palette.

We are now using the RColorBrewer palette “Set3” for the performance plots. An example plot is reproduced here:

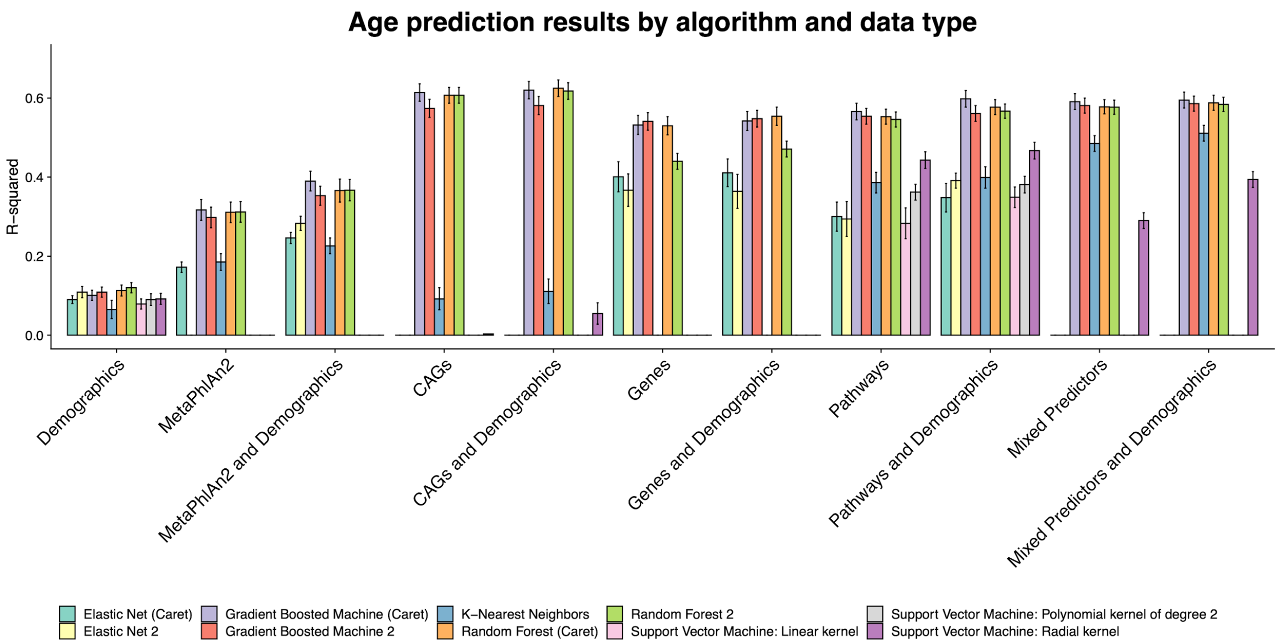

23. I noticed that there were several performance values that were negative in Sup Tab 2 especially for the ElasticNet approach. I think something must have gone wrong. I also noticed several NA performance values for the SVM. Could the authors please explain how this came about?

Thank you for this question – it allowed us to go a bit deeper in the analysis of some of our results. For example, there is indeed a negative performance for R2_test for ElasticNet, and what is interesting to see is that this negative score is driven by a single fold (fold 3) that performed extremely poorly.

It is also interesting to note that this anomaly is found on the same fold for ElasticNet 2, SVM Linear and SVM Polynomial of degree 2, but did not prevent the tree based models (random forest, gradient boosted machine), the KNN and the SVM Radial from performing well. (The SVM Radial performed poorly on the first fold, but not the third one).

This negative performance is only found on the testing set, not on the training set, so the model learned correctly but generalized poorly. What is also interesting to observe is that by looking at the training results and the hyperparameters that were selected, nothing initially suggested that the model trained on this fold would generalize worse than the others: both the training performance and the hyperparameters are similar.

We think there could be two main reasons why this model generalized poorly:

1-It is possible that an outlier in the testing set was predicted with a huge error, which is driving this hugely negative R-Squared score. One way to test it would be to leave out different subsets of the testing set when computing the R-Squared until this negative score disappears to isolate the samples responsible for it. We could follow up with an analysis as to what makes these samples so unpredictable by the linear regression.

2-The problem might not be on the testing set but on the training set. This seems unlikely since this training set shares 89% of its data with the other training sets, but it is possible that this specific combination lead to a poor learning of the regression weights and strong overfitting. One way to test

for that would be to build a correlation matrix between the vector of the weights for each of the 10 models (one for each fold) and to see if the weights learned on the 3rd model differ significantly from the others.

That being said, figuring out why exactly this specific model performed poorly is beyond the scope of this paper. We were only interested in figuring out which model would yield the most robust prediction and we think that the global negative performance of the ElasticNet model sends the right message: it is not always a reliable choice for this specific prediction task.

The NAs performance values for SVM solely come from the predictions using genes. As explained in point 13 (Reviewer 2) , we extracted the genes from the 10 most significant CAGs. For ElasticNet, we took the best 10 CAGs selected by the elastic net. For the GBM, we took the best 10 CAGs selected by the gradient boosted machine. However for the SVM, there is to our knowledge no way to tell which CAGs were the most significant. We could therefore not extract genes, and this is the reason why we used NAs as placeholders for SVMs. This is also true for the Naive Bayes algorithm. Only the elastic nets, the random forests and the gradient boosted machines allowed us to select significant cags and to extract genes from them.

An alternative would have been to use the genes selected by a different algorithm (e.g GBM) to predict the target phenotypes using SVMs and Naive Bayes. Out of consistency we did not do it. Considering the general performance of SVMs and Naive Bayes across the different predictors and targets, we think it is unlikely that they would have performed significantly better than other algorithms using genes as predictors.

To clarify our thinking along these lines to readers, we now include the above text in the Supplementary Text portion of our manuscript.

24. I would make reviewing the paper much easier if the authors could provide page and line numbers and include the figures in the text.

We now provide line and page numbers and have concatenated all the content into a single PDF (with the exception of Excel spreadsheets, which we have kept separate) as well as uploading separate, high resolution figures.

25. The authors write “The exception to this rule was for the Antibiotic Usage phenotype, which primarily had annotated genes, some of which were associated with common pathways of antibiotic resistance, like outer membrane assembly (BamA), DNA repair (XerD), and efflux (YycB).” Please provide some references here. XerD is a recombinase not involved in DNA repair but rather in integration of genetic material such as resistance genes.

We thank the reviewer for pointing out this error, and we have corrected it in the text, writing in the discussion that:

”The exception to this rule was for the Antibiotic Usage phenotype, which primarily had annotated genes, some of which were associated with common pathways of antibiotic resistance or potentially horizontal gene transfer, like outer membrane assembly (BamA), recombination (XerD), and efflux (YycB).”

Reviewer #3: The authors present an analysis of 1570 microbiome samples from 300 infants from four countries. They appear to have two main goals: 1) to characterize the dataset and 2) to show how analytical choices affect the results of that characterization. They show broad variation in prediction accuracy based on modeling choices, and make several insightful recommendations for how practitioners should proceed. They also present interesting results about co-abundance groups that predict infant age, and some insights on the utility of different metagenomic features for this and other purposes.

In general, I think there should be more papers like this with a rigorous analysis of how modeling choices affect results. I recommend this paper for publication, however I do have some comments that I would like to see addressed.

We are grateful the reviewer sees the value in our manuscript and have sought to address all of the points they mentioned.

Major comments:

1. It is concerning that the demographics can be predicted from each other at all. In particular, geography and sex seem tightly correlated. This is a fundamental limitation of the dataset that needs more attention and discussion.

We thank the reviewer for this comment and agree that discussion of the correlation between demographic variables is crucial for this manuscript. This critique encouraged us to look back at our web resource on the "Associations between variables" tab, on which we visualize the relationship between different microbiome and/or demographic features in our study. Below, we reproduce the plot (from the website) for country and sex:

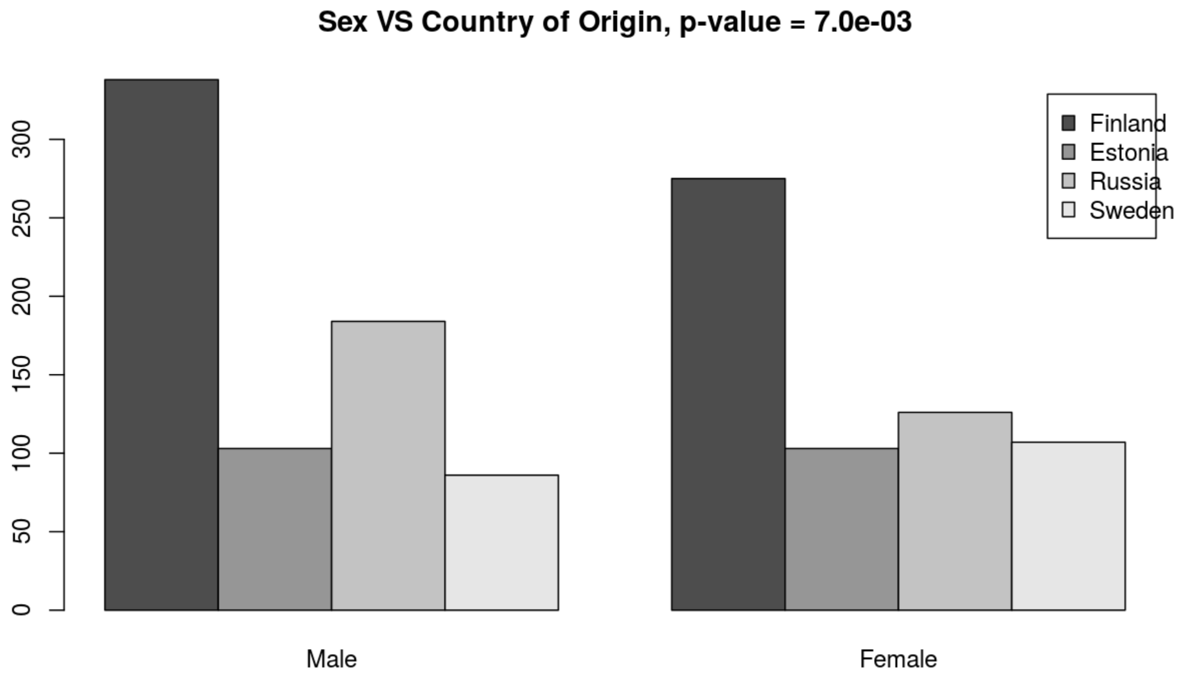

We do not observe a strong correlation between these two features, however we wish to address the reviewer's concern with regards to these data as well as the other demographic features. As such, we now clarify in the results that the relationships between any features – demographic and/or microbiome – can be explored in our web resource:

"Our complete results, as well as a full exploration of the relationships between our demographic variables and cohorts, can be viewed at http://apps.chiragjpgroup.org/ubiome_predictions/. This resource includes the associations between and distributions of features used in training (e.g. age and sex), top microbiome predictors of phenotypes, outcomes for specific models and datatypes, and detailed model training/output information (e.g. sensitivities, specificities, and model hyperparameters)."

Per the request of another reviewer, we additionally have added a Supplementary Figure (Supp Fig 1, reproduced below) demonstrating the results of a principal component analysis (PCA) on the demographic variables (age, sex, country, breastfeeding status, antibiotic usage, and delivery type) colored by cohort. This is to indicate that overall, the cohorts have similar distributions of features and are not highly biased. As we stated previously, though, we strongly encourage readers to investigate this result for themselves at a higher resolution than PCA can provide using the web app.

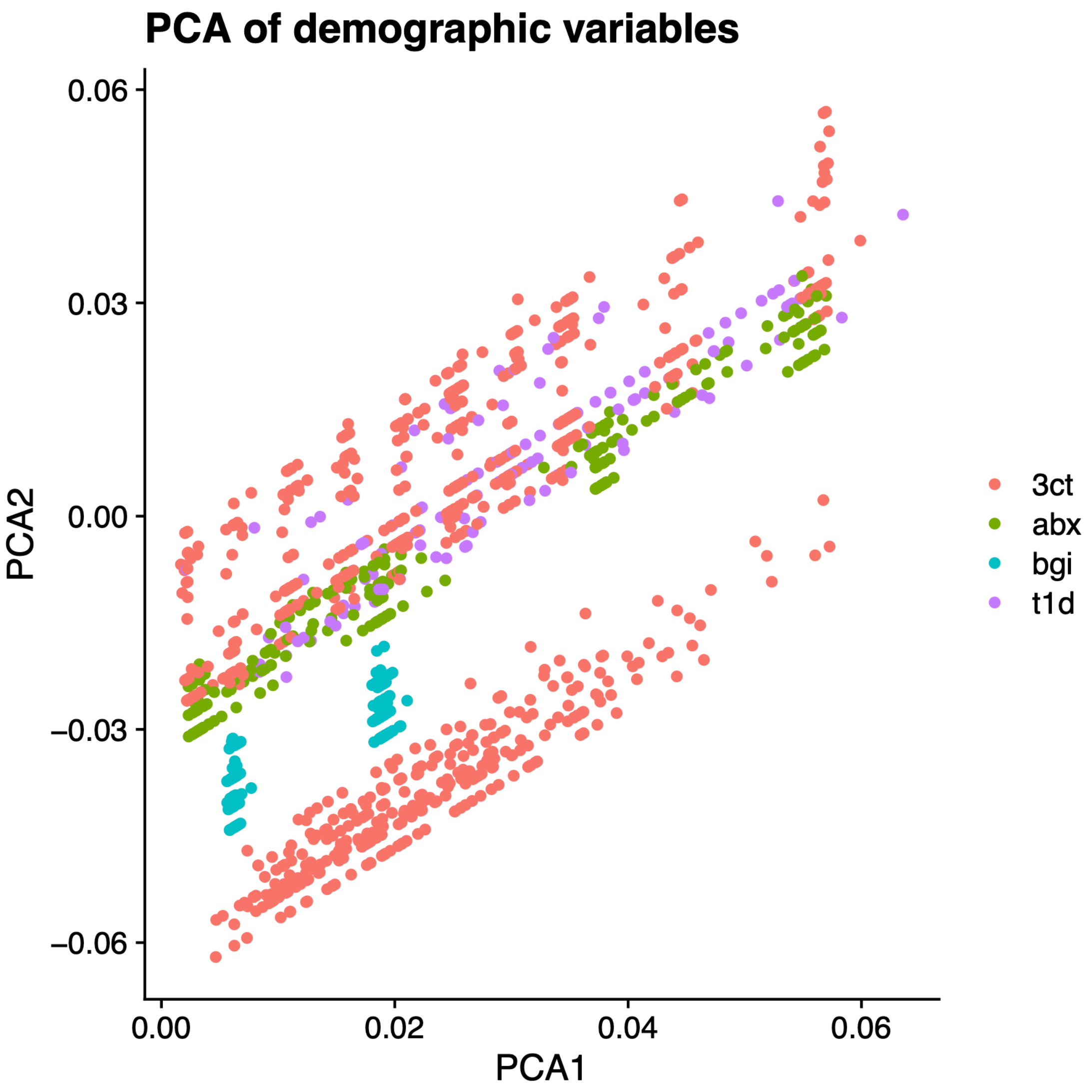

Supplementary Figure 1: Exploratory principal component analysis of all demographic variables, colored by dataset.

2. Of the non-demographic traits only birth-type has a significant increase in accuracy after adding microbiome to the model. This seems particularly interesting as it has a natural causal direction (birth type -> changes in microbiome). I think more attention should be paid to the results of this model, in the same way as for age. Are there genes/taxa/etc that seem to be affected by birth type? Could these have any biological or medical implications?

We agree this is an interesting point for further exploration. Given that genes were the most informative feature for delivery type outside of demographics (we chose not to investigate CAGs as we did for age due to their limited predictive ability), we looked out the annotations of the top genes but were unable to find any interesting biological trends. That said, as we state in multiple points within this document and the paper itself, we hesitated to dive too far into biological explanations derived exclusively lists of gene annotations. As such, while we address these results now, we believe fundamentally they need to be followed up in a future study to be medically or biologically useful.

We specifically mention this point in the results:

"Finally, we sought to further investigate the identified microbiome associations with mode of infant delivery, as our results indicated a substantial increase in prediction accuracy when including microbiome data instead of demographic features alone. While the majority (85/100) of delivery-type associated features had no BioCyc or Prokka-derived annotation, we found a number (3 genes) that mapped specifically to methylation-associated processes. We additionally found two different associated genes to map to the pathway of heparin degradation. Overall, we were unable to identify specific biological trends in the association between these genes and delivery type."

3. My intuition is that the lack of significant effects for the other non-demographic phenotypes seems to be driven more by the high predictive power of the demographics than a lack of signal from the microbiome. It would be interesting to see if these results changed on a more homogeneous sub-cohort, where the predictive power of the demographics would be reduced. This could be as simple as separate male/female analyses.

We agree with the reviewer that this is an important question. To address it, we have included several "controls" in the initial version of the paper.

When we build a model which includes the microbiome as predictor, we also build a model that includes the demographics as predictors along them, as well as a model that predict the phenotype using the sole demographics variables. When the microbiome contains information about the phenotype that is not already encoded in the demographics, the demographics+microbiome model will significantly outperform the demographics_only model. When it does not but the microbiome_only model still performs reasonably well, it suggests that, when used as the sole predictor in a model, the microbiome is possibly merely finding a way to predict the demographics to which the model does not have access (e.g country of origin) before using this confounder as a predictor. We are basing this approach on published methods.(McNamee 2005)

Running two separate analysis would divide our already limited sample size by two and would multiply the number of computing resources we would need. On top of that, This is another reason why we decided on using these controls rather than splitting the cohort to run different sub-analyses.

Based on our results and the comparison between the model and the different controls, we estimate that it is unlikely that the predictive power of sex is shadowing the significance of the effects of the microbiome. As we show, for phenotypes such as age, delivery type or country of origin, we found that adding the microbiome as a predictor significantly improved the prediction over a model based on the sole demographics variables.

4. I would not say that the authors have “successfully predicted sex from the microbiome” (p9). The vast majority of the predictive power for sex is coming from the fact that it is tightly coupled to geography, and while the additional contribution of the microbiome is statistically significant it is also small. This claim should be closer to “we have identified genes/taxa/etc that are correlated with sex”.

We thank the reviewer for this comment and have since softened this statement and included the recommended text by the reviewer. We have removed references to “successfully predicting sex,” stating instead in the summary that:

“Our pipeline was able uncover robust microbial associations with phenotype”

And in the discussion that:

“We reported biological associations for our most robust phenotype-associated findings, including age, delivery type, sex, and antibiotic usage.”

5. To deal with the fact that some individuals contribute multiple samples, the authors enforce the constraint that all samples from an individual are in the same fold. This is fine, but there are other ways to deal with this (choosing one sample per person, learning individual-specific profiles and correcting for them). This is an unexplored aspect of potential variation in the results. Since variation in results due to analytical choices is the main theme of the paper, that choice should at least be highlighted before the method section.

We agree with the reviewer that this is an important point that we should have specified in the paper. Reviewer 2 made a similar comment, and we responded in the following way:

“We agree with the reviewer that ideally we should have kept individuals from the same study in the same folds. However, because of the limited number of individuals and samples per individuals in some of the studies, imposing this extra constraint would have left us with highly imbalanced folds in terms of sample sizes. For imbalanced classes, some classes would not have been represented in some folds. This is the reason why we decided that keeping the samples from each participant in the same fold was a fair trade-off.

That being said, we definitely agree that the issue of confounding variables is a big one. This is the very reason why we built “control” models where we used solely demographics variables as predictors. If most of our predictive power came from the demographics variables, which include country of origin, we would not see a significant difference between the predictive accuracy obtained on the “demographics only” model and the models that leverage microbiome information. As observed in Table 1, there was indeed

no significant difference for antibiotics usage and for exclusive breastfeeding. On contrary, significant differences were observed for age, delivery type and sex, which shows that the extra predictive accuracy comes from the microbiome variables and cannot be explained by cohort differences."

We have additionally sought to clarify this analytical choice in the results:

"For each model, we used 10x10 fold nested cross validation (Supp Figure 3), specifying that all samples from a given individual were present within the same fold"

Minor comments:

6. Perhaps the ordering of the results should be changed. The abstract, introduction and discussion all heavily focused on the question of predictive consistency, but the results are primarily about the biological implications of the analysis until the last section.

We thank the reviewer for this comment and have now re-ordered the results. The formerly last section, with the concordance analysis, is now closer to the front (coming second) such that the model evaluation and biological results are kept separate. Additionally, the classification results (regarding antibiotic usage, breastfeeding status, delivery type, country, and sex) are now at the head of the paper. We believe this effort has resulted in a more logical and deductive flow for our work.

7. Author affiliation 6 is missing a department

We now clarify that the relevant department is the "Lewis-Sigler Institute for Integrative Genomics."

8. The concordance of feature importance between methods seems particularly relevant, perhaps Supp Fig 6 should be a main Figure. However, I can't seem to access the Supp Figures, did the author's forget to upload them? I see only supp tables 1-5.

We sincerely apologize for this lapse and have confirmed the supplementary figures are now uploaded with this submission. Additionally, in reordering our results we have made Supplementary Figure 6 a main figure, as we agree it is a critical finding to communicate.
