# Supplementary material for: A systematic machine learning and data type comparison yields metagenomic predictors of infant age, sex, breastfeeding, antibiotic usage, country of origin, and delivery type
Source: PLoS Comput Biol. 2020 May 11;16(5):e1007895. doi: 10.1371/journal.pcbi.1007895 (PMC7241849; doi:10.1371/journal.pcbi.1007895)
Supplement: Attachment — Submitted filename: review_revised_version.pdf [file pcbi.1007895.s022.pdf]

Reviewer comments for the revised version of
"A systematic machine learning and data type comparison yields metagenomic predictors of infant age, sex, breastfeeding, antibiotic usage, country of origin, and delivery type"

I appreciate that the authors went through a great effort to address the comments I and the other reviewer's raised. They were able to allay most of my concerns. However, I think there are a number of issues persisting that need to be resolved before I can full-heartedly recommend publication of their work.

- The authors managed to convince me that the controls they used for the demographics are likely sufficient and I appreciate that they now include some summary statistics for the demographics in the paper. Could the authors please give some examples what demographic variables were predictive especially for the antibiotic usage and exclusive breastfeeding phenotypes? I think that by itself is an interesting result and even beyond what demographics boosted prediction in addition to the microbiome predictors e.g. for delivery type and country of origin. I also thank the authors for providing an exploratory analysis of their data. I think they should state more clearly that there is indeed some stratification by study. More technically could they provide a more descriptive legend in the PCA plot and furthermore describe what transformation they applied to the data before conducting the PCA?
- With respect to my suggestion of using a stratified cross-validation approach to, I can follow their argument of potentially ending up with very imbalanced folds. Could the authors, instead extend the PCA analysis to the gene/pathway/CAGs (with appropriate standardization of the features). The issue is not only with the demographics leaking through the microbiome features but also that cohorts may be identifiable by themselves due to batch effects i.e. the samples having been processed using the same protocol within cohorts.
- In Supplementary Table 7 could the authors explain what the two numbers in every table cell mean? Are these the significantly associated taxa and CAGs, respectively?
- In response to 19: Thanks for the clarification. I think the BioCyc Tier 3 annotation procedure may be limited in low homology settings because it's relying on pairwise DIAMOND comparisons. Profile HMM based approaches like Pfam or eggNOG are well established approaches even for function transfer from remote homologs. I suggest to add this to the discussion.
- In response to 23, I thank the authors for looking into this issue. Given the almost bizarre values of R^2 -1751585582200.95+-1524893838954.27 in one instance (SVM polynomal kernel) and recurring negative value across different data types (CAGs, Taxa, Demographics), I think the authors should really try to identify those outlier samples. For each samples in every test fold they should be able to extract the predicted value, which might identify suspicious cases without requiring complex further analysis.
