# Supplementary material for: A systematic machine learning and data type comparison yields metagenomic predictors of infant age, sex, breastfeeding, antibiotic usage, country of origin, and delivery type
Source: PLoS Comput Biol. 2020 May 11;16(5):e1007895. doi: 10.1371/journal.pcbi.1007895 (PMC7241849; doi:10.1371/journal.pcbi.1007895)
Supplement: Attachment — Submitted filename: response_to_reviewers.pdf [file pcbi.1007895.s023.pdf]

04/16/2020

Dear Drs. Papin, Segata and reviewers:

We thank you and the Reviewers for the feedback and further opportunity to improve our manuscript. As before, we addressed reviewer concerns point-by-point below in blue text.

Thank you again for your consideration, and we look forward to a favorable response.

On behalf of the co-authors,

Chirag Patel and Aleksander Kostic

Reviewer 1:

1) I would still consider narrowing down the number of models tested (while moving some to the supp material) so the paper can be more concise and (yet again) more educating to the reader in terms of types of models and their expected behavior.

   We thank the reviewer for this comment, as a core goal of our manuscript is for it to instruct readers regarding modeling practices in the microbiome discovery and research. It is for this reason, however, that we hesitate to move all the models to the supplement, as we believe a key strength of our work is the fact that the main figures summarize such a broad array techniques transparently, including those that are less common in microbiome analyses but frequently used in the machine learning community (e.g. SVMs) to show the vast array of findings possible when analyzing a large microbiome dataset.

   That said, we agree that our paper could be more educational in terms of the types of models and their expected behavior. As such, we have elected to include a supplementary text section describing, at a high level, the various models tested, their characteristics, and their similarities and differences. We hope that this section will be useful to readers who are less familiar with the modeling strategies outlined in our manuscript.

   We write (in the Supplementary section titled ”A brief overview of our selected machine learning tools”):

   “We employ a range of modeling strategies in our manuscript. Here, we describe the broad characteristics, similarities, and differences between them.

   The simplest method we use is an Elastic Net (EN). An EN is a combination of Ridge and Lasso regression -- it is a linear model designed to aid in variable selection, where highly-outcome-associated features (e.g. species, pathways, or genes) are identified among a large set. Given their nature, ENs are only capable of capturing linear-relationships between dependent and independent variables, meaning that while their results are at times easier to

interpret biologically and statistically than other “black-box” methods, they can potentially overlook valid non-linear structures within the data.

We additionally employed Random Forests (RFs), ensemble learning methods that are popular in microbiome analyses. Each random forest is a combination of decision trees fit to the data, where each the nodes of each tree represent classes or outcomes, and the branch points represent features in the data that distinguish different classes or outcomes. Individual decision trees are fit on a subsample of the data -- the “Forest” results from the combination (weighted averaging) of these individual trees. RFs are able to capture non-linear effects, it is difficult to carry out statistical inference on their output due to their “black box” nature (e.g., information on how increasing a certain variable modulates the dependent variable is not as easily attainable as for Elastic Nets).

Gradient-Boosted-Machines (GBMs) are another ensemble learning approach that we use. GBMs leverage “gradient boosting” -- often applied to decision trees -- where “weak-learners” (poorly performing trees) are optimized for increased performance by, for a given tree, increasing the weights on difficult-to-classify/predict features in the training set and lowering the weights on the easy-to-classify outcomes. The next tree is built with these weights, therefore resulting in a model sensitive to variation across outcomes. Like an RF, GBMs too are often treated as black boxes, so despite being able to capture non-linear effects, they can also be difficult to interpret (e.g., information on how increasing a certain variable modulates the dependent variable is not as easily attainable as for Elastic Nets).

We additionally used 3 different Support Vector Machines (SVMs), with a linear, polynomial, and radial kernel. SVMs attempt to project data into an N-dimensional space where it can be spatially separated into groups. The kernel in question corresponds to how the data are projected into this new coordinate plane; depending on the kernel, an SVM can capture non-linear effects. Again, inference on the individual variables is not easily attainable.

Finally, we additionally used, but due to their weak performance, are not discussed heavily K-nearest-neighbors and Naive Bayes classifiers. The former is a simple supervised method where outcomes are grouped into a given number of input clusters based on separation (e.g. Euclidean distance). The latter is a simple classifier based on Bayes theorem, which assigns the posterior probability an individual is in a group based on given input features (e.g. species abundances), which are assumed to be completely independent.”

We reference this section in the main text by writing:

“For a high-level overview of these modeling strategies, please see the Supplementary Text.”

Reviewer 3:

1) The authors managed to convince me that the controls they used for the demographics are likely sufficient and I appreciate that they now include some summary statistics for the demographics in the paper. Could the authors please give some examples what demographic

variables were predictive especially for the antibiotic usage and exclusive breastfeeding phenotypes? I think that by itself is an interesting result and even beyond what demographics boosted prediction in addition to the microbiome predictors e.g. for delivery type and country of origin.

We thank the reviewer for their interest in this question and for giving us the opportunity to talk about our results in more detail. We agree that these results are interesting and should be discussed in greater depth.

In short, both antibiotic usage and breastfeeding status were well-predicted by country of origin. Age at collection was also a strong predictor for the latter, however it is worth noting that its relative importance varied depending on algorithm choice. We now mention this in the Results section:

“Other demographic variables were highly predictive: for example, the maximum AUC for demographic variables along predicting antibiotic usage was 0.786+-0.03. Most notably, country of origin and age at collection were the most predictive of our other phenotypes (though their relative importance varied depending on algorithmic choice).”

We also have provided the findings on our Shiny application, and we show screenshots below:

Antibiotics usage:
For antibiotics usage it can be seen in figure A below that similar performances were obtained using linear and non-linear algorithms when the predictors used were the demographics variables (group of columns on the left). We will therefore communicate the features importance from the elastic net, as it is more interpretable. They can be found in figure B. Living in Sweden or Russia was, for example, negatively associated with antibiotics usage.

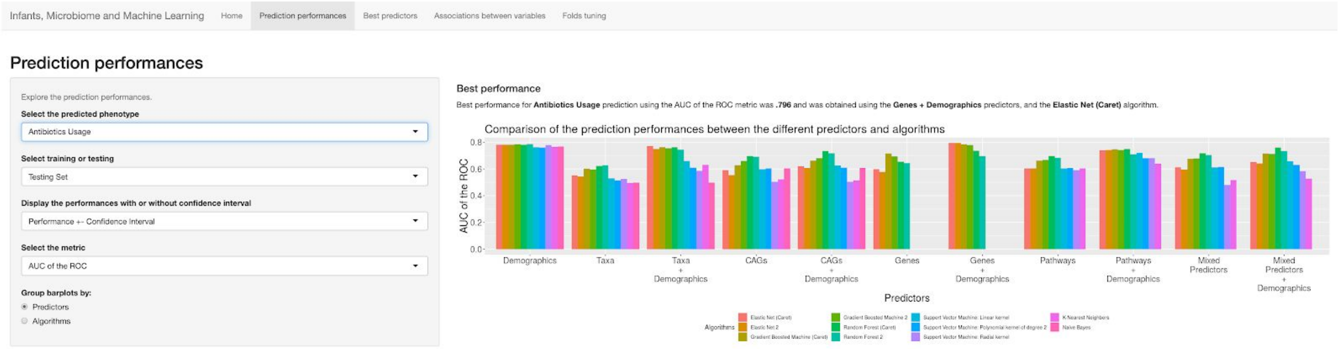

	Demographics	Taxa	Taxa + Demographics	CAGs	CAGs + Demographics	Genes	Genes + Demographics	Pathways	Pathways + Demographics	Mixed Predictors	Mixed Predictors + Demographics
Elastic Net (Caret)	.783+-.014	.551+-.017	.771+-.014	.592+-.017	.621+-.017	.600+-.017	.796+-.013	.604+-.017	.740+-.014	.611+-.016	.652+-.016
Elastic Net 2	.782+-.013	.543+-.017	.751+-.014	.556+-.017	.608+-.016	.578+-.017	.796+-.013	.604+-.016	.743+-.015	.596+-.016	.642+-.016
Gradient Boosted Machine (Caret)	.781+-.013	.601+-.016	.764+-.014	.628+-.017	.664+-.016	.716+-.015	.785+-.013	.662+-.016	.747+-.014	.675+-.016	.717+-.015
Gradient Boosted Machine 2	.784+-.013	.598+-.018	.755+-.015	.660+-.016	.681+-.016	.695+-.015	.780+-.013	.669+-.016	.743+-.014	.678+-.016	.714+-.015
Random Forest (Caret)	.781+-.013	.623+-.016	.763+-.014	.698+-.016	.735+-.015	.654+-.016	.738+-.015	.699+-.015	.751+-.015	.717+-.015	.760+-.014
Random Forest 2	.786+-.013	.628+-.016	.745+-.015	.691+-.016	.719+-.015	.644+-.016	.698+-.015	.685+-.016	.711+-.015	.705+-.016	.734+-.015
Support Vector Machine: Linear kernel	.763+-.014	.530+-.018	.661+-.016	.600+-.017	.626+-.016	NA+-NA	NA+-NA	.606+-.016	.722+-.015	.612+-.017	.658+-.016
Support Vector Machine: Polynomial kernel of degree 2	.761+-.015	.514+-.015	.610+-.017	.605+-.016	.610+-.016	NA+-NA	NA+-NA	.608+-.017	.682+-.016	.615+-.017	.630+-.017
Support Vector Machine: Radial kernel	.780+-.013	.526+-.016	.587+-.017	.504+-.016	.504+-.015	NA+-NA	NA+-NA	.592+-.016	.682+-.015	.482+-.024	.583+-.017
K-Nearest Neighbors	.767+-.014	.497+-.013	.632+-.016	.523+-.016	.516+-.016	NA+-NA	NA+-NA	.605+-.017	.639+-.016	.519+-.016	.527+-.017
Naive Bayes	.768+-.014	.500+-.001	.500+-.001	.604+-.015	.607+-.015	NA+-NA	NA+-NA	NA+-NA	NA+-NA	NA+-NA	NA+-NA

Figure A: Performance of the different algorithms and predictors for prediction of “antibiotics usage”.

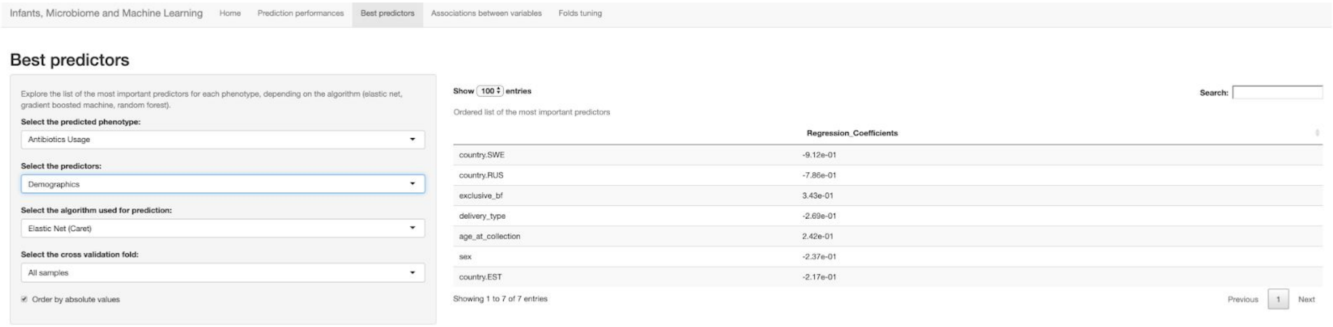

	Regression_Coefficients
country.SWE	-9.12e-01
country.RUS	-7.86e-01
exclusive_bf	3.43e-01
delivery_type	-2.69e-01
age_at_collection	2.42e-01
sex	-2.37e-01
country.EST	-2.17e-01

Figure B: Features importance extracted from the elastic net predicting antibiotics usage as a function of the demographic variables.

Exclusive breastfeeding:

For exclusive breastfeeding, it can be seen in figure C that, using demographics predictors only, the non-linear algorithm GBM performed significantly better than the non-linear algorithm elastic net. We will therefore answer the reviewer’s question based on this algorithm’s features importance, which can be found in figure D. Age was the most important predictor, followed by country of origin (Estonia and Sweden) and antibiotics usage. Interestingly, age was not detected as an important predictor by the linear model that is the elastic net (see figure E), which suggests that it was the combination between age and other demographics variables that leveraged its predictive power.

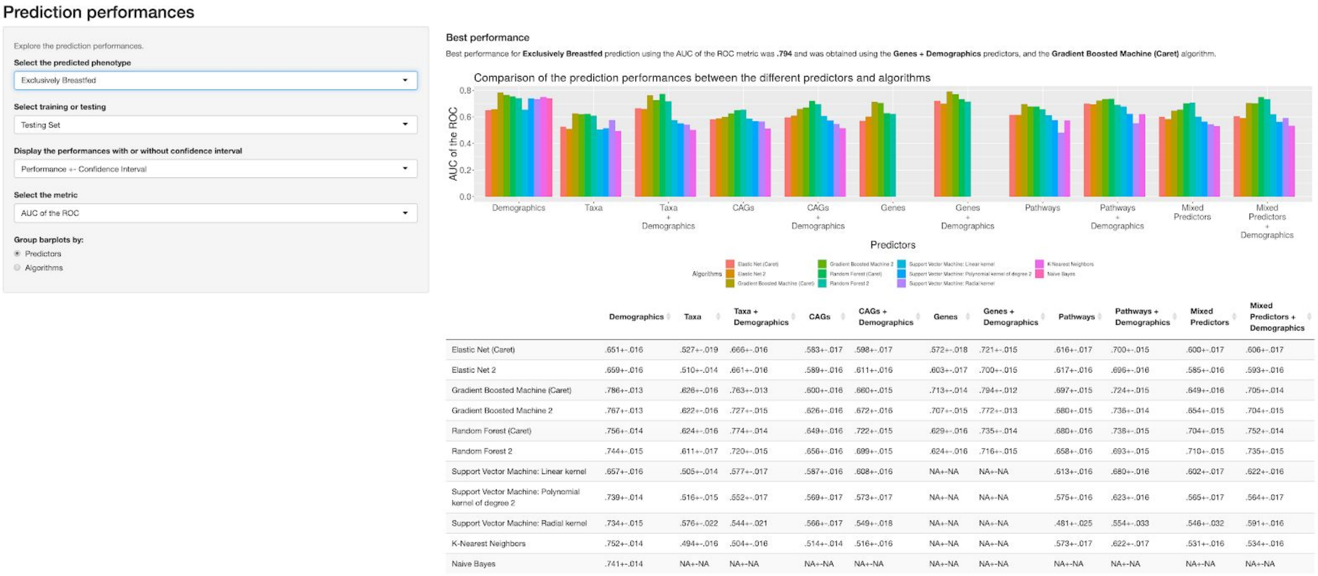

	Demographics	Taxa	Taxa + Demographics	CAGs	CAGs + Demographics	Genes	Genes + Demographics	Pathways	Pathways + Demographics	Mixed Predictors	Mixed Predictors + Demographics
Elastic Net (Caret)	.651+-.016	.527+-.019	.666+-.016	.583+-.017	.598+-.017	.572+-.018	.721+-.015	.616+-.017	.700+-.015	.600+-.017	.606+-.017
Elastic Net 2	.659+-.016	.510+-.014	.661+-.016	.589+-.016	.611+-.016	.603+-.017	.700+-.015	.617+-.016	.696+-.016	.585+-.016	.593+-.016
Gradient Boosted Machine (Caret)	.786+-.013	.626+-.016	.763+-.013	.600+-.016	.660+-.015	.713+-.014	.794+-.012	.697+-.015	.724+-.015	.649+-.016	.705+-.014
Gradient Boosted Machine 2	.767+-.013	.622+-.016	.727+-.015	.626+-.016	.672+-.016	.707+-.015	.772+-.013	.680+-.015	.736+-.014	.654+-.015	.704+-.015
Random Forest (Caret)	.756+-.014	.624+-.016	.774+-.014	.649+-.016	.722+-.015	.629+-.016	.735+-.014	.680+-.016	.738+-.015	.704+-.015	.752+-.014
Random Forest 2	.744+-.015	.611+-.017	.720+-.015	.656+-.016	.699+-.015	.624+-.016	.716+-.015	.658+-.016	.693+-.015	.710+-.015	.735+-.015
Support Vector Machine: Linear kernel	.657+-.016	.505+-.014	.577+-.017	.587+-.016	.608+-.016	NA+-NA	NA+-NA	.613+-.016	.680+-.016	.602+-.017	.622+-.016
Support Vector Machine: Polynomial kernel of degree 2	.739+-.014	.516+-.015	.552+-.017	.569+-.017	.573+-.017	NA+-NA	NA+-NA	.575+-.016	.623+-.016	.565+-.017	.564+-.017
Support Vector Machine: Radial kernel	.734+-.015	.576+-.022	.544+-.021	.566+-.017	.549+-.018	NA+-NA	NA+-NA	.481+-.025	.554+-.033	.546+-.032	.591+-.016
K-Nearest Neighbors	.752+-.014	.494+-.016	.504+-.016	.514+-.014	.516+-.016	NA+-NA	NA+-NA	.573+-.017	.622+-.017	.531+-.016	.534+-.016
Naive Bayes	.741+-.014	NA+-NA	NA+-NA	NA+-NA	NA+-NA	NA+-NA	NA+-NA	NA+-NA	NA+-NA	NA+-NA	NA+-NA

Figure C: Performance of the different algorithms and predictors for prediction of “antibiotics usage”

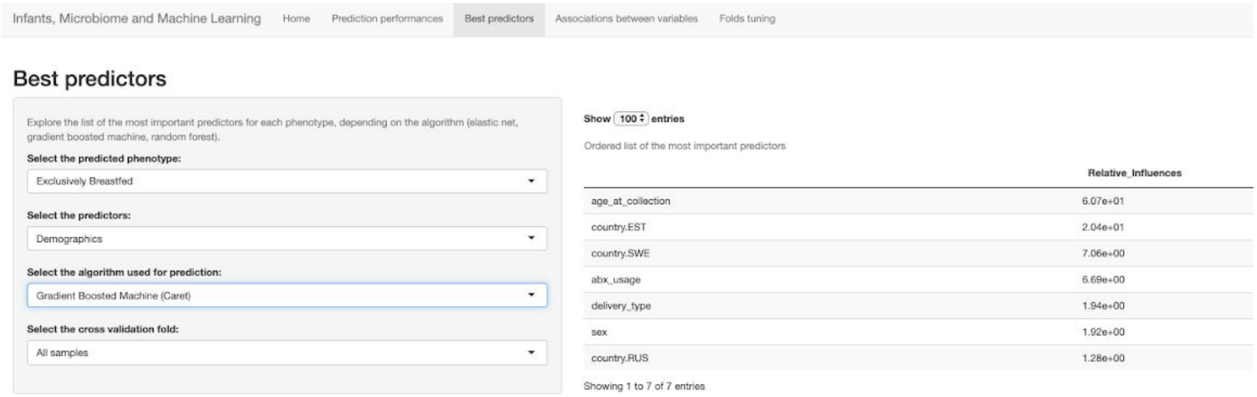

Figure D: Feature importance extracted from the GBM that predicted antibiotics usage using the demographics variables.

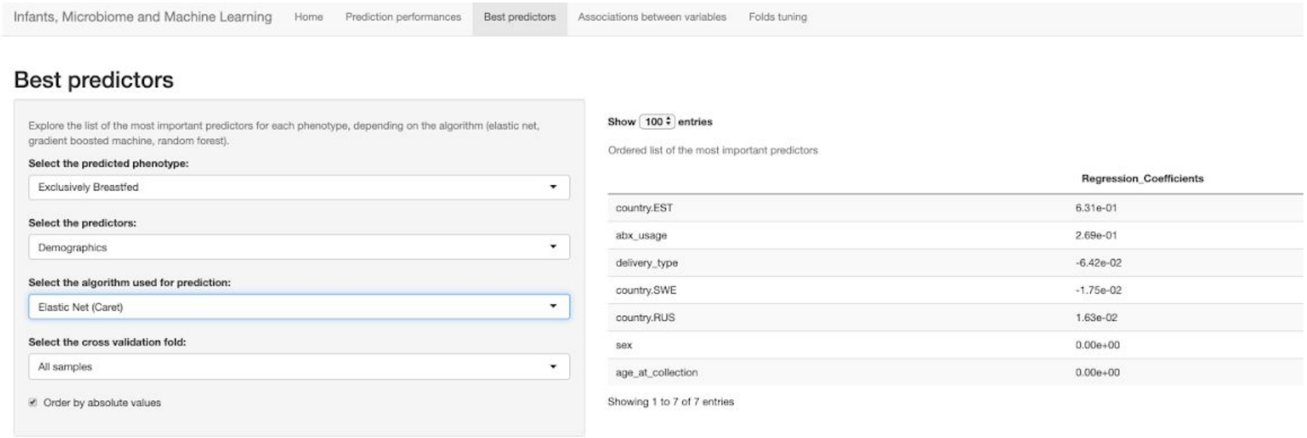

Figure E: Feature importance extracted from the elastic net that predicted antibiotics usage using the demographics variables.

Delivery type:

For delivery type, the best performance was obtained using an elastic net and the genes along the demographics as predictors, so we are displaying the features importance for this model in figure F. We can for example observe a negative association with antibiotics usage, exclusive breastfeeding and Russia, and a positive association with age and Sweden.

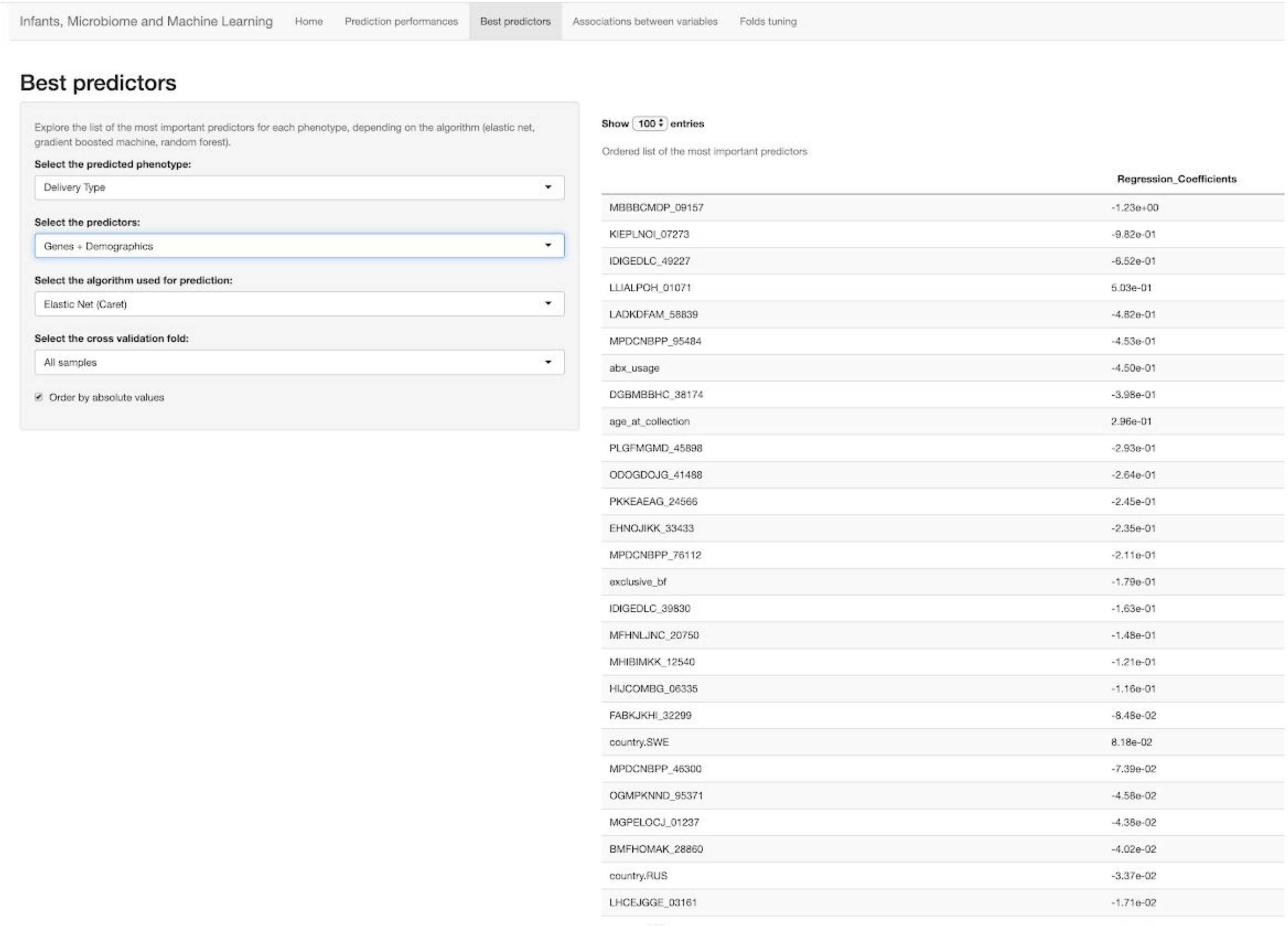

Figure F: Features importance for delivery type

Country of origin:

For country of origin, the best performance was obtained using the genes along the demographics as predictors, and a GBM as the algorithm. We report the features importance for this model in Figure G.

Notable demographics features of importance are exclusive breastfeeding, sex and antibiotics usage.

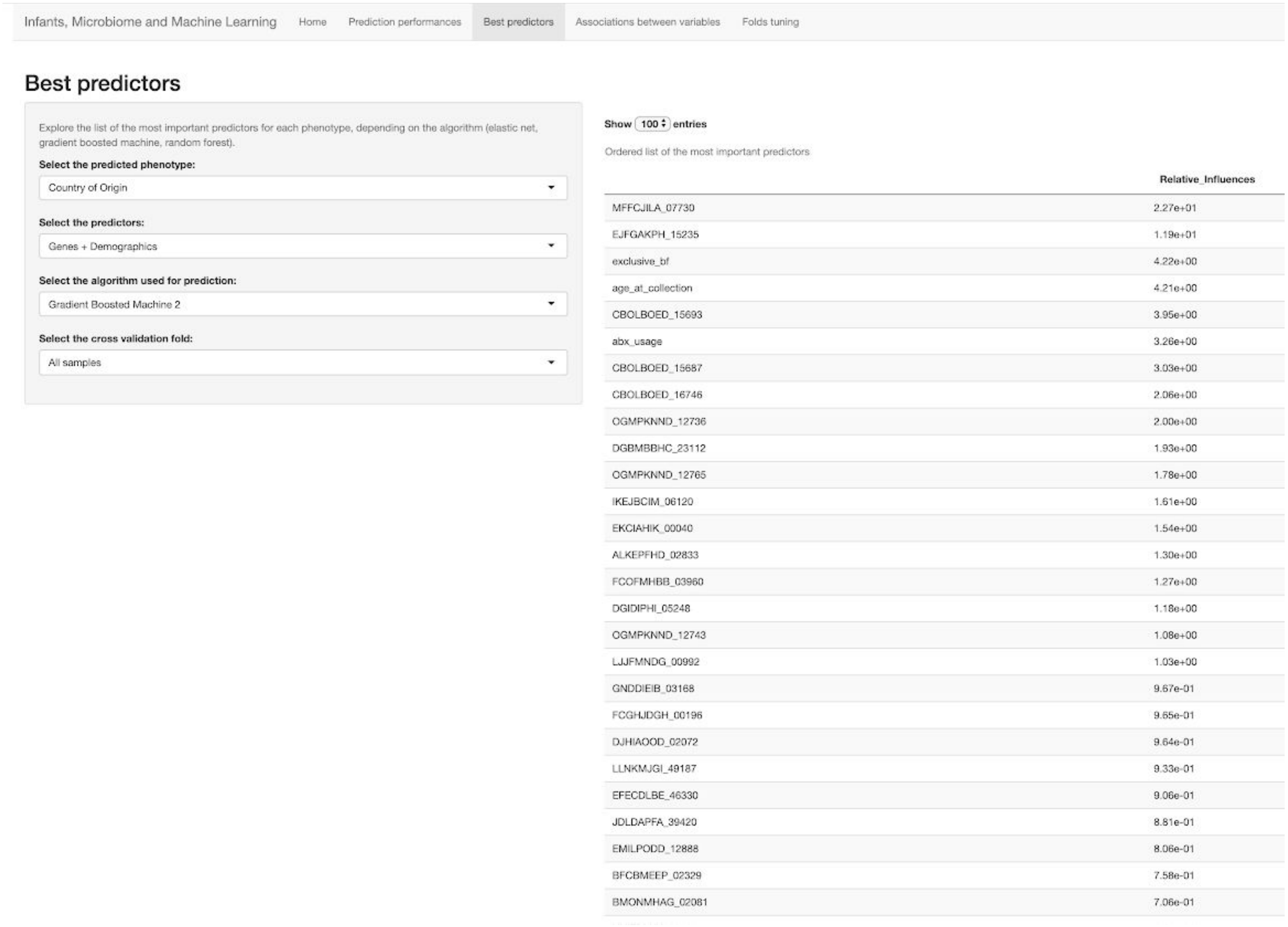

Figure G: Features importance for country of origin

We hope that the shiny app we deployed will be a convenient tool for the readers to further investigate the association between the predicting and predicted phenotypes that are of a particular interest to them (http://apps.chiragjpgroup.org/ubiome_predictions/).

2) I also thank the authors for providing an exploratory analysis of their data. I think they should state more clearly that there is indeed some stratification by study. More technically could they provide a more descriptive legend in the PCA plot and furthermore describe what transformation they applied to the data before conducting the PCA?

We now mention in the results that we do see stratification by study, writing:

“It is worth nothing that preliminary principal component analysis did identify some stratification between datasets on the basis of metadata alone and by microbiome data type, particularly between the Swedish (Bäckhed_2015) cohort and the others (Supp Fig 1).”

We additionally describe in the methods exactly how we computed our PCA:

“We executed our Principal Component Analysis using R’s prcomp() function, scaling input columns to have unit variance.”

Finally, we have adjusted the legend in the PCA plot (reproduced below in beneath Point 3) to clarify exactly which datasets/studies the colors correspond to.

3) With respect to my suggestion of using a stratified cross-validation approach to, I can follow their argument of potentially ending up with very imbalanced folds. Could the authors, instead extend the PCA analysis to the gene/pathway/CAGs (with appropriate standardization of the features). The issue is not only with the demographics leaking through the microbiome features but also that cohorts may be identifiable by themselves due to batch effects i.e. the samples having been processed using the same protocol within cohorts.

We have now extended our PCA to the metaphlan, pathway, and CAG abundance data, standardizing features beforehand. We additionally thank the reviewer for noting that we should be scaling input vectors prior to running PCA, as we had neglected to do so upon initial revision. Due to issues with computational scale, and the fact that the CAGs are a superset of the gene data, we did not do this for the gene abundances. The plots are included as part of Supplementary Figure 1 and reproduced below for convenience.

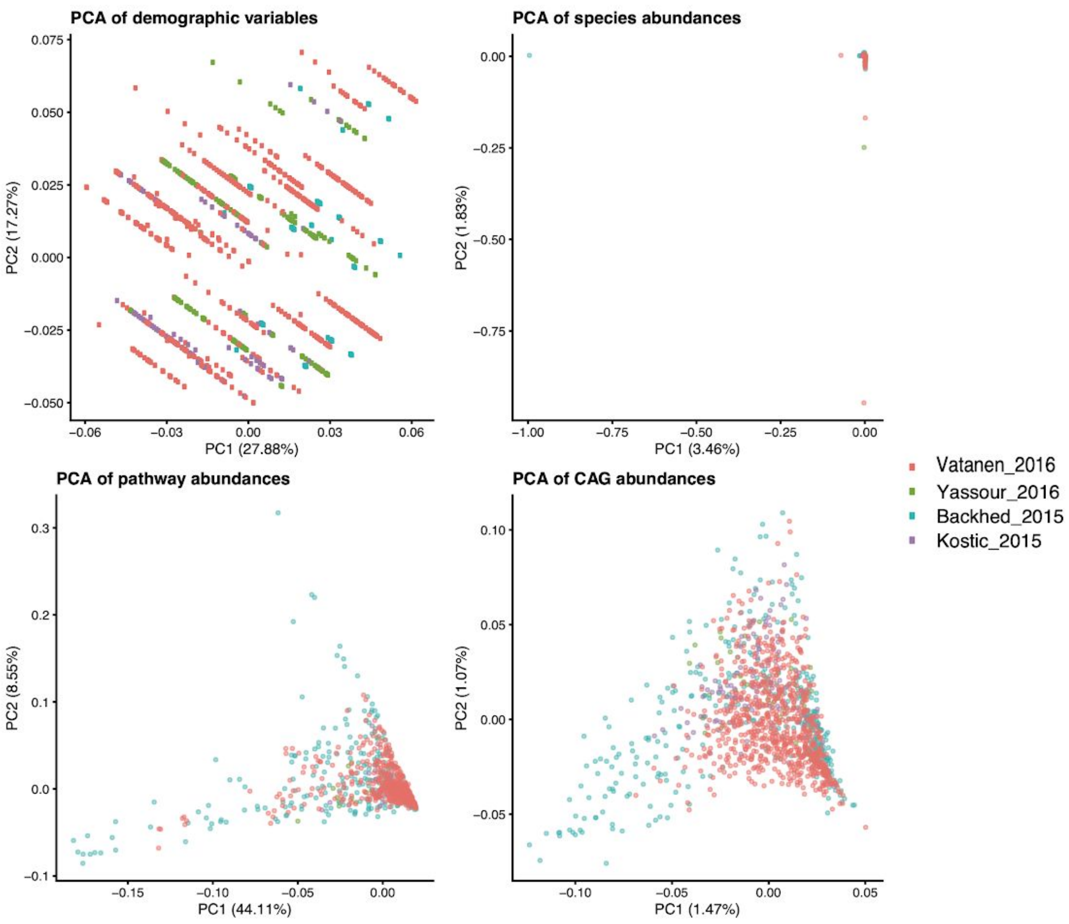

4) In Supplementary Table 7 could the authors explain what the two numbers in every table cell mean? Are these the significantly associated taxa and CAGs, respectively?

We agree our initial version of this table was unclear; as such, we have modified it appropriately. We wanted to provide more than the raw number of significant features, because the total number of features is not the same for each predictors category (e.g Taxa vs CAGs). We now realize how unclear it can be, so we now communicate the absolute number of significant predictors, along the percentage it represents with respect to the total number of predictors. We have reproduced the new table below:

**Target**	**Taxa (N=714)**	**Pathways (N=1282)**	**CAGs (N=8292)**
Age	37 (5.18%)	1010 (78.78%)	623 (7.51%)
Sex	0 (0.00%)	4 (0.31%)	0 (0.00%)
Country of Origin	130 (18.21%)	548 (42.75%)	340 (4.10%)
Exclusively Breastfed	366 (51.26%)	1 (0.08%)	2 (0.02%)
Antibiotics Usage	0 (0%)	183 (14.27%)	17 (0.21%)
Delivery type	0 (0%)	29 (2.26%)	3 (0.04%)

Supplementary Table 7: All results for fold "0" (all samples): Bonferroni corrected associated and statistically significant predictors in a univariate regression/classification context. Percentage in parentheses is fraction of total predictors tested that were statistically significant.

5) In response to 19: Thanks for the clarification. I think the BioCyc Tier 3 annotation procedure may be limited in low homology settings because it's relying on pairwise DIAMOND comparisons. Profile HMM based approaches like Pfam or eggNOG are well established approaches even for function transfer from remote homologs. I suggest to add this to the discussion.

We agree with this point and now explicitly mention it in the discussion, writing:

"In future work, alternative pathway annotation methods that are not reliant on homology alone (e.g. pFam, eggNOG) should be tested in a similar approach to ours.(Huerta-Cepas et al. 2019; Finn et al. 2014)"

6) In response to 23, I thank the authors for looking into this issue. Given the almost bizarre values of R^2 -1751585582200.95+-1524893838954.27 in one instance (SVM polynomial kernel) and recurring negative value across different data types (CAGs, Taxa, Demographics), I think the authors should really try to identify those outlier samples. For each samples in every test fold they should be able to extract the predicted value, which might identify suspicious cases without requiring complex further analysis.

We thank the reviewer for pointing out this issue. First, to be clear, we believe that this phenomenon is driven by two things:

#1: It happens that one of the folds fails a negative R-Squared result emerges. When this anomaly is observed for several algorithms on a specific fold, and only this fold, it indeed suggests that (at least) one outlier is present in this fold, and that the models generalize so poorly to this outlier that it drives the overall performance down. An example of this happening is the prediction of age using all the predictors (Predictors: Mixed + demographics). For the Elastic Nets, SVMs (linear and polynomial kernels), all folds yielded a decent performance, except for the fold 3. In contrast, the tree based algorithms and the K-Nearest Neighbors algorithms yielded decent performances on all folds. What is most likely happening is that one of the samples in fold 3 has an extremely large/low value for one of the predictors. For the elastic net and the SVM (Linear and polynomial kernels), this value is extrapolated and drastically increases/decreases the age predicted. In contrast, for tree based methods, this value will be interpreted as belonging in a bucket (e.g CAG_value > 3) and will not overly affect the age predicted. K-Nearest Neighbors will also be robust to this kind of outliers, as it will simply find the closest neighbors, even if the outlying abnormal value means the distance will be large.

The predictor with the abnormal and outlying value is most likely a CAG, since the models using other subsets of predictors (e.g Taxa only, Genes only, Pathways only) did not suffer from this issue. It is not certain though, as other folds also failed for the taxa only model, which was not repeated in the Mixed predictors model.

Fundamentally, we are not convinced that identifying these outlier samples is within the immediate scope of this manuscript and would add value to our overall goal, identifying vibration in model results across a range of prediction and classification strategies. It is well known that the microbiome has immense variation between samples -- as such, the presence of outliers will likely not surprise individuals within the field, and identification of particular samples as such will only demonstrate that they exist. Certainly, biologically investigating why a microbial sample is or isn't an outlier in a cohort is interesting, but again that is not within our scope. Furthermore, the choice to remove or keep outliers in a model is of immense debate and is yet another factor that can lead to diverging results.

All that said, identifying algorithms and data that enhance robustness to outliers is an important feature (which we saw to be the case for tree based methods such as GBM and RF, and not in ENs or SVMs) of a modeling strategy, so we now mention this point in the discussion, writing:

"At times, even different implementations of the same algorithms (i.e. Random Forests and GBMs) yielded differing predictive results. We observed some with negative prediction performances (Supplementary Text). We believe these poor performances arose in some cases for outliers present in the training folds, and that algorithms robust to the presence of such outliers (GBMs, RF) were not strongly affected, unlike ENs and SVMs.

#2: Some algorithms fail to generalize for every fold. The R-Squared of -1751585582200.95+-1524893838954.27 for age prediction using a SVM with polynomial kernel and Taxa+Demographics as predictors is one of these. The fold performances ranged from -.038 to -18000521537053.598. This is unlikely to be driven by outliers. Rather, the algorithm consistently fails to generalize. We additionally reference this point in the discussion:

“Finally, negative performances could also be observed on every testing fold for some models (e.g SVM with polynomial kernel), which suggests that the model simply fails to generalize.”
